# Poly-(L-homoarginine) for the non-invasive treatment of endophthalmitis

**DOI:** 10.1016/j.mtbio.2025.101931

**Published:** 2025-06-02

**Authors:** Ting Hua, Tianzi Zhang, Yi Tang, Shuo Wang, Guowenlie Gao, Chunsheng Xiao, Pengqi Wan, Hong Wu

**Affiliations:** a Department of Ophthalmology, The Second Hospital of Jilin University, Changchun, 130041, China; b Affiliated Hospital of Inner Mongolia University for the Nationalities, Inner Mongolia, 028000, China; c Key Laboratory of Polymer Ecomaterials, Changchun Institute of Applied Chemistry, Chinese Academy of Sciences, Changchun, 130022, China

**Keywords:** Antibacterial polymers, Bacterial infection, Endophthalmitis, Polypeptides, Topical instillation

## Abstract

Bacterial endophthalmitis (BE) is a severe eye infection that often leads to vision loss or blindness. The conventional therapeutic approach entails the administration of intravitreal antibiotic injections, a procedure that is associated with significant discomfort for the patient. Here, we present a kind of poly-(L-homoarginine) (C_6_PLL_10_-Gua) as a non-invasive alternative for effectively eliminating bacteria in endophthalmitis. The synthesized C_6_PLL_10_-Gua effectively kills various bacteria by disrupting cell membranes, without causing bacteria resistance over 14 generations. More importantly, C_6_PLL_10_-Gua demonstrated significant tissue permeability both *in vivo* and *in vitro*. This kind of C_6_PLL_10_-Gua eye drops were capable of effectively traversing the ocular barrier to access the posterior segment of the eye. After being instilled into the conjunctival sac, C_6_PLL_10_-Gua enabled rapid (<30 min) and prolonged (>24 h) distribution in the retina via a noncorneal pathway. Furthermore, in a MRSA induced endophthalmitis rat model, topical instillation of C_6_PLL_10_-Gua showed significant therapeutic effect, which presenting a promising non-invasive therapeutic approach for managing endophthalmitis.

## Graphical abstract

A poly-(L-homoarginine) was readily synthesized via a combination of ring opening polymerization (ROP) of *N*-carboxyanhydride (NCA) monomers and post-polymerization modification, which exhibited effective eye permeability and excellent antibacterial property.

**Figure:**
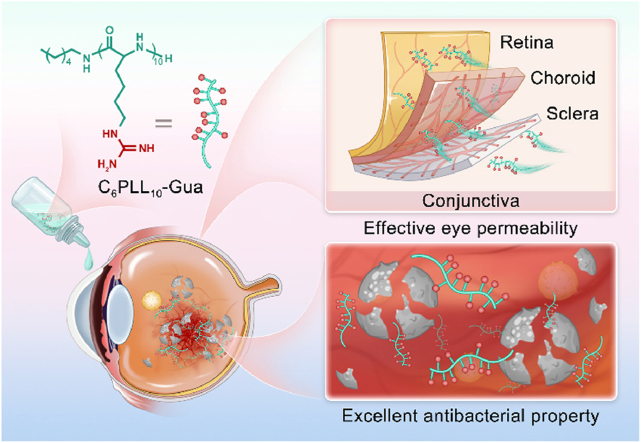

## 1 Introduction

Bacterial endophthalmitis (BE) is a severe eye infection that can cause blindness, occurring in about 0.1 % of cataract surgeries [1,2]. The posterior segment of the eye, particularly the fundus area, serves as the primary site of endophthalmitis, characterized by tissue irreplaceability and irreversible damage [[3], [4], [5], [6]]. In clinical practice, the standard treatment involves the administration of intraocular antibiotic injections, which is invariably accompanied by a vitrectomy procedure [3,7]. The overuse and misuse of antibiotics, coupled with the widespread occurrence of multidrug-resistant bacteria, has significantly hindered the efficacy of monotherapy with antibiotics [[8], [9], [10], [11], [12]]. Besides, the antibiotics-associated hemorrhagic occlusive retinal vasculitis has been documented in certain cases of bacterial endophthalmitis following intravitreal vancomycin administration, resulting in significantly diminished visual acuity [13,14]. Due to the limited effectiveness of certain antibiotics against specific bacteria and the inadequate ocular penetration of antibiotic eye drops, repeated injections become necessary, leading to time-consuming and costly treatments for patients [15]. Therefore, developments of alternative antibacterial therapeutics for BE are imperative.

It is widely recognized that, in contrast to intravitreal injections, eye drops administration offer a non-invasive and on-demand method of intraocular delivery [16,17]. However, the distinctive physiological characteristics of the eye present challenges in administering antibiotic eye drops to the posterior chamber. Consequently, the development of drugs with enhanced ocular barrier permeability is crucial for their clinical application in the treatment of endophthalmitis. It is well-documented that, arginine-rich cell-penetrating peptides (CPPs) have been proved effective in membrane permeation, helping chemical drugs, nucleic acids, and macromolecules to across physiologic barriers, including bacterial, algal, and mammalian cell membranes, human skin, and the blood-brain barrier, especially the eye barrier [[18], [19], [20], [21], [22], [23], [24]]. The guanidine group in arginine is regarded as a crucial element for the permeability of these peptides. It forms bidentate hydrogen bonds with negatively charged components (such as sulfate, carboxylate, and phosphate) on the cell membrane and facilitates the penetration of polymers into the interior of tissues [[25], [26], [27]]. Inspired by these studies eye drops formulated with cationic poly (α-amino acids) modified with guanidinium groups may offer highly effective, non-invasive, real-time therapeutic options for drug delivery to the posterior cavity.

**Scheme 1:**
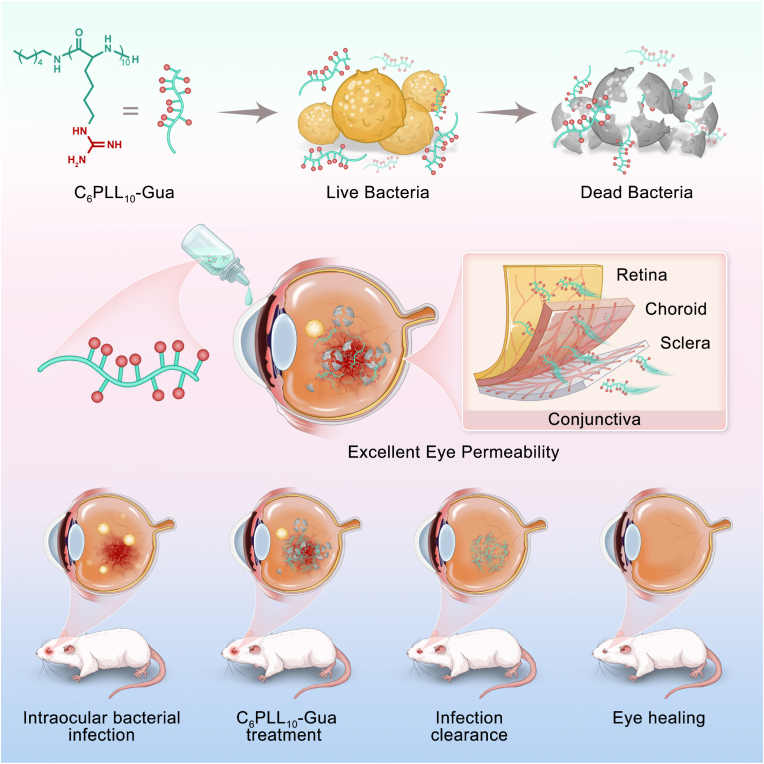
Schematic illustration of the chemical structure of C_6_PLL_10_-Gua. C_6_PLL_10_-Gua can effectively destroy the integrity of bacterial membrane and has excellent penetration ability of eye tissue. C_6_PLL_10_-Gua shows effective therapeutic effect in rat endophthalmitis model.

Herein, we synthesized a kind of poly-(L-homoarginine) (C_6_PLL_10_-Gua) as an eye drops for the non-invasive treatment of bacterial endophthalmitis (Scheme 1). The optimal polymer, C_6_PLL_10_-Gua demonstrates effective *in vitro* antibacterial ability and low hemolysis. The antibacterial mechanism reveals that C_6_PLL_10_-Gua can damage bacteria membrane and will not induce bacterial resistance. More significantly, C_6_PLL_10_-Gua demonstrated effective permeability across ocular barriers both *in vivo* and *in vitro*. Upon administration into the conjunctival sac, it facilitated rapid distribution within less than 30 min and sustained presence for over 24 h, achieving peak concentrations in the retina at 6 h through a non-corneal route. *In vivo* anti-infective property of C_6_PLL_10_-Gua eye drop has also been demonstrated in a MRSA-induced rat endophthalmitis model.

## 2 Results and discussion

### 2.1 Synthesis of C_6_PLL_10_-Gua

**Fig. 1:**
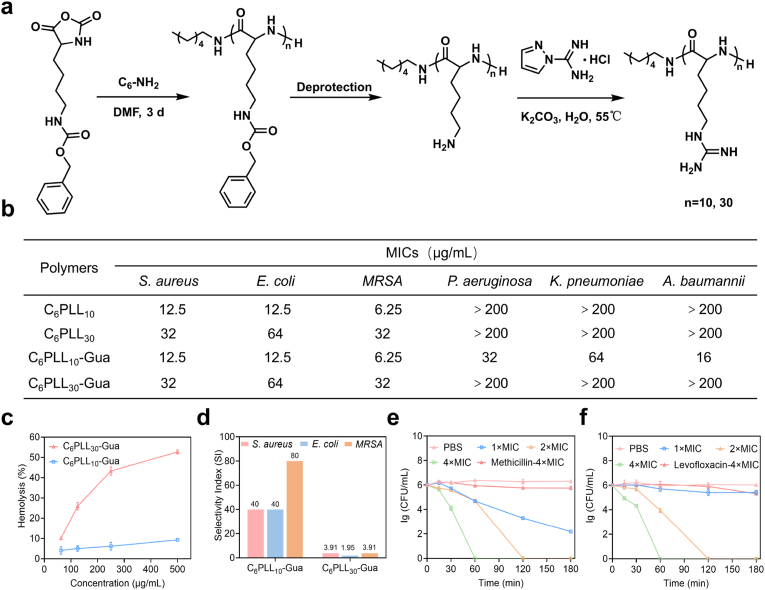
a) The synthetic route of C_6_PLL_n_-Gua. b) The MIC values of synthetic polymers against different bacteria. c) Hemolytic activity of C_6_PLL_10_-Gua and C_6_PLL_30_-Gua. d) Selectivity index (HC_10_/MIC) of polymers against *S. aureus*, *E. coli*, and MRSA. *In vitro* killing kinetics of C_6_PLL_10_-Gua, methicillin, and levofloxacin. CFUs of e) *S. aureus* and f) *E. coli* after cultivation with C_6_PLL_10_-Gua or C_6_PLL_30_-Gua at a range of concentrations (1 × , 2 × , and 4 × MIC) and cultivation with methicillin or levofloxacin at 4 × MIC.

The detailed synthetic pathway of C_6_PLL_n_-Gua was illustrated in Fig. 1a. First, *N*-hexylamine was used to initiate ring opening polymerization (ROP) of *ε*-benzyloxycarbonyl-_L_-lysine *N*-carboxyanhydride (ZLL-NCA) monomers (Fig. S1 and Table S1). After deprotection of carbobenzyloxy (Cbz) in 33 % HBr/CH_3_COOH, C_6_PZLL_n_ were obtained. According to the ^1^H NMR spectrum (Fig. S2 and S3), the degrees of polymerization (DP) of C_6_PLL_n_ were 10 and 30, which was calculated by the integral ratio of characteristic peak a (the chemical shift at *δ* = 0.88 ppm is attributed to the terminal −CH_3_ group of the alkyl chain) to characteristic peak b (the chemical shift at *δ* = 4.12 ppm is attributed to the methyl group present in main polymer chain). C_6_PLL_n_ underwent additional modification via the reaction of its amine groups with 1H-pyrazole-1-carboxamidine hydrochloride, resulting in the synthesis of guanidinium-functionalized C_6_PLL_n_, briefly named C_6_PLL_n_-Gua. As shown in Fig. S4 and S5, the successful synthesis C_6_PLL_n_-Gua was validated by the emergence of characteristic peaks c (*δ* = 3.06 ppm, corresponding to the methylene protons in −C*H*_2_NHC (=NH)NH_2_). Furthermore, the GPC result (Table S1) also indicate that C_6_PLL_n_-Gua was successfully synthesized with low *Ð* value of 1.03 and 1.18, respectively.

### 2.2 *In vitro* antibacterial and hemolytic activities

To assess the antibacterial properties of C_6_PLL_n_ and C_6_PLL_n_-Gua, we determined their MICs against a range of bacterial strains. The detailed MIC values are listed in Fig. 1b. C_6_PLL_10_-Gua, with a lower degree of polymerization, demonstrated superior antibacterial efficacy compared to C_6_PLL_30_-Gua. This enhanced activity may be attributed to the peptidoglycan layer of the bacterial cell wall, which likely impedes the penetration of high molecular weight polymers, thereby diminishing their antibacterial effectiveness [28,29]. Both C_6_PLL_10_-Gua and C_6_PLL_10_ exhibit effective antibacterial activities against *S. aureus*, *E. coli*, and MRSA, with MICs of 6.25 μg/mL against MRSA. C_6_PLL_10_-Gua is significantly more effective than C_6_PLL_10_ against other resistant bacteria. The MICs of C_6_PLL_10_-Gua for *P. aeruginosa*, *K. pneumoniae*, and *A*. *baumannii* were determined to be 32, 64, and 16 μg/mL, respectively. This may be attributed to the increased electrostatic interaction between the polymer and the bacteria due to the guanidinium modification compared to C_6_PLL_10_ [30,31]. Concurrently, strains isolated from the conjunctival sac of clinical cataract patients and *E*. *faecalis* were also tested. As shown in Table S2, C_6_PLL_10_-Gua exhibited significant antibacterial activity against *S. wardii*, *S. epidermidis*, and *E*. *faecalis*, with the MICs of 4, 8, and 12.5 μg/mL, respectively. The above results indicate that C_6_PLL_10_-Gua has good antibacterial activity.

To evaluate the biocompatibility of the polymers, we further tested the hemolytic toxicity of the polymers. As shown in Fig. 1c, the hemolysis rate of C_6_PLL_10_-Gua is 9.3 % at the concentration of 500 μg/mL. In contrast, C_6_PLL_30_-Gua exhibits significant hemolytic toxicity (at a concentration of 62.5 μg/mL, the hemolysis rate is 10 %). This potentially attributable to higher degree of polymerization and increased number of guanidine-modified side chains, which induced higher interaction with cell membranes, resulting in elevated cytotoxicity [[32], [33]], [[32], [33]]. The selectivity index (SI) was further calculated by comparing HC_10_ with MIC (HC_10_/MIC) to provide a measured prokaryote vs mammalian cell selectivity. As illustrated in Fig. 1d, C_6_PLL_10_-Gua displayed a much higher SI values than C_6_PLL_30_-Gua, with a SI was 80 for MRSA, and 40 for both *S*. *aureus* and *E*. *coli*. Due to the higher SI value as well as potent antibacterial activity, C_6_PLL_10_-Gua was selected as the optimal antibacterial polymer for further evaluation.

Furthermore, the bactericidal kinetics of C_6_PLL_10_-Gua against bacteria was examined. (Fig. 1e and f). At 2 × MIC, complete eradication of *S. aureus* and *E. coli* was achieved within 2 h. In contrast, samples treated with methicillin or levofloxacin still exhibited viable bacteria within the same period. Furthermore, the bactericidal activity of C_6_PLL_10_-Gua was shown to be dose-dependent, as increasing the concentration from 2 × to 4 × MIC reduced the time required for entirely bacterial clearance from 2 to 1 h. However, even after an extended duration of up to 3 h, methicillin and levofloxacin failed to achieve a 99 % eradication rate of bacteria. The enhanced bactericidal kinetics of C_6_PLL_10_-Gua positions it as a potential therapeutic agent for the management of bacterial infections.

### 2.3 Antibacterial mechanism of C_6_PLL_10_-Gua

**Fig. 2:**
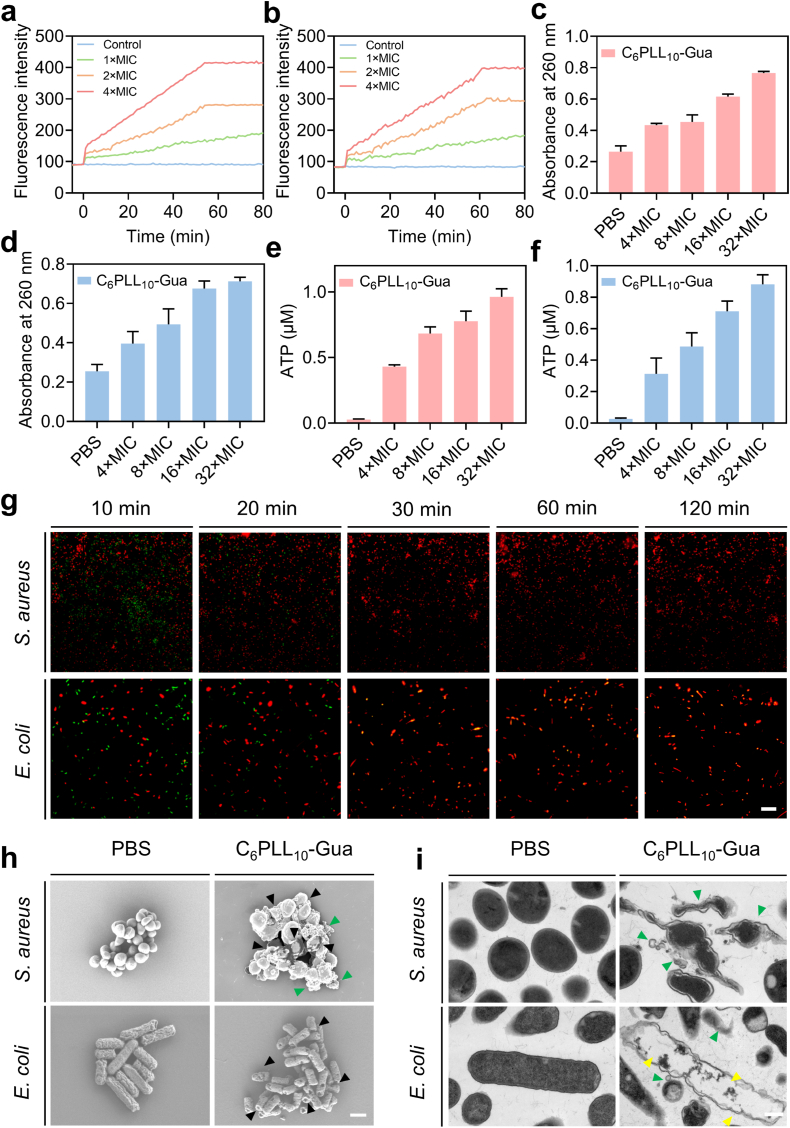
Cytoplasmic membrane permeabilization of *S. aureus* a) and *E. coli* b) following incubation with C_6_PLL_10_-Gua at different concentrations. Absorbance ratios at 260 nm of the supernatant in *S. aureus* c) and *E. coli* d) suspension treated with PBS or C_6_PLL_10_-Gua for 2 h at different concentrations as specified (bacterial density: 2 × 10^9^ CFU/mL). ATP contents in the supernatants of *S. aureus* e) and *E. coli* f) suspension treated with PBS or C_6_PLL_10_-Gua at different concentrations. g) Fluorescence images of bacteria treated with 2 × MIC of C_6_PLL_10_-Gua for different. Scale bar: 1 μm. h) SEM images of *S. aureus* or *E. coli* after different treatments. Black arrow: collapse and rupture of the bacterial membrane, green arrow: cytoplasm flowed out of the bacteria. Scale bar: 1 μm. i) TEM images of *S. aureus* or *E. coli* after different treatments. Yellow arrow: vacuole of bacteria, green arrow: cytoplasm flowed out of the bacteria. Scale bar: 0.5 μm. (For interpretation of the references to colour in this figure legend, the reader is referred to the Web version of this article.)

To investigate the antibacterial mechanism of C_6_PLL_10_-Gua, we used propidium iodide (PI) as a probe to assess bacterial membrane permeability. The binding of PI to DNA, resulting in red fluorescence, indicates damaged integrity of the bacterial cell membrane [34]. A significant dose-dependent cytoplasmic membrane permeabilization activity in *S. aureus* (Fig. 2a) and *E. coli* (Fig. 2b) were observed in the presence of C_6_PLL_10_-Gua. Then, we assessed bacterial rupture by detecting leakage of cytoplasmic components, *e.g*., ATP and DNA after C_6_PLL_10_-Gua treatment. A substantial efflux of cytoplasmic constituents was discovered in all instances of bacteria following 2 h incubation with C_6_PLL_10_-Gua at a 4 × MIC concentration (Fig. 2c-f).

The membrane-damaging capability of C_6_PLL_10_-Gua was assessed using live/dead staining. SYTO 9 green dye stains nucleic acids in both intact and damaged membranes, while PI penetrates only damaged membranes to bind DNA and emit red fluorescence [[35], [36], [37]]. As shown in Fig. 2g, after incubation with C_6_PLL_10_-Gua at 4 × MIC, *S. aureus* or *E. coli* showed more red fluorescence over time. The statistical diagrams of semi-quantitative fluorescence analysis intensity in each group showed in Fig. S7 and S8. After processing for more than 30 min, the red fluorescence ratio is 100 %. After C_6_PLL_10_-Gua was co-incubated with C_6_PLL_10_-Gua at 2 × MIC for 12 h, we collected *S. aureus* and *E. coli*, observed scanning electron microscopy (SEM) photographed and transmission electron microscopy (TEM) ultrathin sectioned images. As shown in Fig. 2h and i, the bacteria in the PBS-treated group maintained a healthy morphology. In the PBS-treated group of *S. aureus* and *E. coli*, plump bacterial contours and clear cell walls, bacterial plasma membranes, filled bacterial cytoplasm were observed. At 1 × MIC concentration, after incubation with C_6_PLL_10_-Gua, the bacteria showed obvious collapse and rupture of the bacterial membrane, a large amount of cytoplasm has flowed out of the bacteria, presenting a vacuolar appearance. Thus, the material can effectively destroy cell membrane integrity. All these results suggest that the C_6_PLL_10_-Gua kill the bacteria by breaking the cytomembrane, which is analogous to the most reported natural antimicrobial peptides (AMPs) [38].

Repeated exposure of microorganisms to sublethal antibiotic doses can lead to drug resistance [39]. To evaluate the efficacy of C_6_PLL_10_-Gua in preventing resistance, *S. aureus* was exposed to sub-MIC levels of C_6_PLL_10_-Gua, with MIC values assessed over 14 generations. Methicillin was used as a control. Fig. S6 illustrates that C_6_PLL_10_-Gua maintained stable MIC values, whereas methicillin's MIC values rose 16-fold after 14 generations. This indicates that *S. aureus* develop minimal resistance to C_6_PLL_10_-Gua, making it advantageous for treating *S. aureus* infections due to its distinct antibacterial mechanism.

### 2.4 Ocular permeability of C_6_PLL_10_-Gua-FITC *in vivo* and *in vitro*

**Fig. 3:**
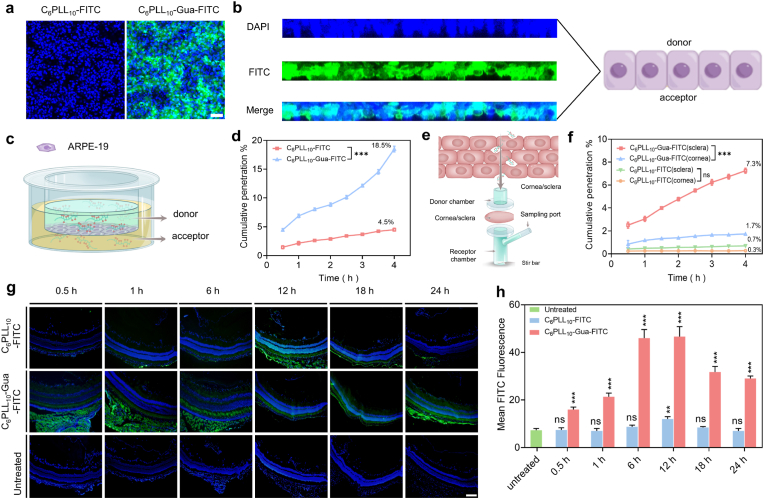
a) Fluorescence images of ARPE-19 cells were aligned towards the donor side. Scale bar, 100 μm. b) The penetration depth of C_6_PLL_10_-Gua-FITC through the monolayer. The nuclei were stained with DAPI (blue). c) Schematic illustration of the *in vitro* model of ARPE-19 cell monolayer. d) Cumulative amount of transported C_6_PLL_10_-FITC or C_6_PLL_10_-Gua-FITC in the acceptor side. The cumulative penetration of C_6_PLL_10_-Gua-FITC group was higher than that in C_6_PLL_10_-FITC group and the difference was statistically significant (∗∗∗*p* < 0.001 vs C_6_PLL_10_-FITC). e) Illustration of Franz diffusion cells using a rabbit's posterior segment as the membrane. f) Cumulative penetration rates by group. The cumulative penetration of C_6_PLL_10_-Gua-FITC (sclera) group was higher than that in C_6_PLL_10_-Gua-FITC (cornea) group and the difference was statistically significant (∗∗∗*p* < 0.001 vs C_6_PLL_10_-Gua-FITC (cornea)). There was no significant difference between C_6_PLL_10_-FITC (sclera) group and C_6_PLL_10_-FITC (cornea) group (^ns^*p* > 0.05). g) Intraocular distribution of C_6_PLL_10_-FITC and C_6_PLL_10_-Gua-FITC in retina at different time points. Scale bar, 200 μm. h) Semi-quantitative analysis of fluorescence intensity in each retina at different time points. Fluorescence intensity means were calculated using ImageJ, and statistical significance was assessed with One-way ANOVA and Dunnett's test (n = 3). At different times of C_6_PLL_10_-Gua-FITC group was higher than that in untreated group, and the difference was statistically significant (∗∗∗*p* < 0.001). There was no significant difference between C_6_PLL_10_-FITC group and untreated group (^ns^*p* > 0.05). (For interpretation of the references to colour in this figure legend, the reader is referred to the Web version of this article.)

The ocular permeability of the material is crucial for the treatment of endophthalmitis. To further study the permeability of polymer within the ocular, C_6_PLL_10_ or C_6_PLL_10_-Gua labeled with FITC (green) during the delivery process *in vivo* and *in vitro*. The ARPE-19 cell monolayer model was developed to assess the permeability of C_6_PLL_10_-Gua-FITC across the posterior ocular barriers (Fig. 3c). As shown in Fig. 3a, there was nearly no green fluorescent signal in C_6_PLL_10_-FITC treated monolayer, but following treatment with C_6_PLL_10_-Gua-FITC, a noticeable fluorescence was detected in the monolayer. From a lateral perspective (Fig. 3b), C_6_PLL_10_-Gua-FITC demonstrated the ability to permeate the entire monolayer. Furthermore, C_6_PLL_10_-Gua-FITC traversed the monolayer in a time-dependent fashion within a 4 h period (Fig. 3d). Following the incubation period, the basolateral side exhibited a presence of up to 18.5 % of C_6_PLL_10_-Gua-FITC, representing a 4.1-fold increase compared to C_6_PLL_10_-FITC.

Subsequently, the Franz diffusion cell apparatus, utilizing isolated rabbit corneas and sclera, was employed as a standardized methodology to assess the penetration capabilities of C_6_PLL_10_-Gua-FITC and C_6_PLL_10_ -FITC against the cornea and sclera [16,40]. In this experiment, C_6_PLL_10_-Gua -FITC and C_6_PLL_10_-FITC was introduced into the donor chamber, and the materials transmitted to the receptor chamber were quantified by measuring fluorescence signals at various time intervals (Fig. 3e). As shown in Fig. 3f, the fluorescence of C_6_PLL_10_-FITC (sclera) group and C_6_PLL_10_-FITC (cornea) group were very weak, and the fluorescence of C_6_PLL_10_-Gua-FITC (sclera) group and C_6_PLL_10_-Gua-FITC (cornea) group were obviously higher. Notably, C_6_PLL_10_-Gua -FITC exhibited a highest fluorescence intensity, 3.8 times greater than in the cornea at 4 h, and the fluorescence intensity of C_6_PLL_10_-Gua-FITC (sclera) increased a five-fold times from 1 h to 4 h. The findings indicate that C_6_PLL_10_-Gua is likely to traverse the ocular barrier via a non-corneal pathway.

To assess the *in vivo* ocular barrier penetration capability of the material, we examine its distribution throughout the entire eyeball of mice at various time points following administration via eyedrops. After topical instillation, C_6_PLL_10_-FITC solution exhibited minimal penetration into the ocular tissues of the mice, evidenced by the absence of a detectable fluorescence signal within the interior of intact eyeballs (Fig. 3g). The fluorescence of C_6_PLL_10_-FITC is concentrated in the conjunctiva. Conversely, the topical administration of C_6_PLL_10_-Gua-FITC into the conjunctival sac resulted in a pronounced fluorescent signal, predominantly observed in the posterior segment of the retinal sections. The C_6_PLL_10_-Gua-FITC was observed to accumulate extensively in the periocular tissue within the 0.5–6 h timeframe, functioning as a reservoir to facilitate the prolonged absorption of C_6_PLL_10_-Gua-FITC to the retina. Based on the semi-quantitative findings (Fig. 3h), the fluorescence intensity of C_6_PLL_10_-Gua-FITC in the retina reached its maximum at 6 h post-instillation, with a retention duration exceeding 24 h. The findings suggest that C_6_PLL_10_-Gua demonstrates significant ocular permeability, thereby offering a promising therapeutic approach for the management of endophthalmitis.

### 2.5 Treatment of bacterial endophthalmitis

**Fig. 4:**
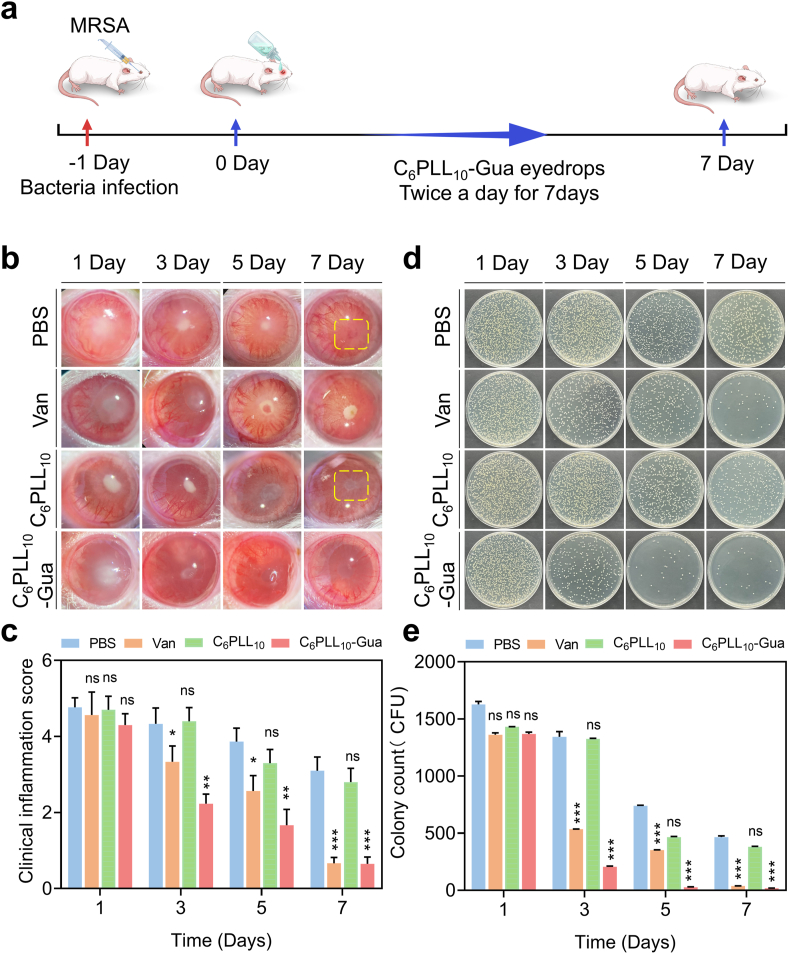
a) Protocol for evaluating the efficacy of C_6_PLL_10_-Gua treatment at different time points after establishing the rat MRSA endophthalmitis model. b) Typical Eye slit-lamp photos on days 1, 3, 5, and 7 in different treatments, yellow dashed rectangle region: purulent exudates and fibrin presented in the anterior segment of the eye. c) Rats eye inflammation score. The clinical score of c on days 3, 5, 7 of C_6_PLL_10_-Gua group and van group was lower than that in PBS group, and the difference was statistically significant (∗*p* < 0.05, ∗∗*p* < 0.01, ∗∗∗*p* < 0.001). There was no significant difference between C_6_PLL_10_ group and PBS group (^ns^*p* > 0.05). d) Images depicting bacterial cultures on agar plates derived from the corresponding aqueous humor at various time intervals. e) Bacterial colony count of rat eyes. The colony count of endophthalmitis on days 3, 5, 7 of C_6_PLL_10_-Gua group and van group was lower than that in PBS group, and the difference was statistically significant (∗∗∗*p* < 0.001). There was no significant difference between C_6_PLL_10_ group and PBS group (^ns^*p*>0.05). (For interpretation of the references to colour in this figure legend, the reader is referred to the Web version of this article.)

Endophthalmitis presents with a rapid onset, is associated with a poor prognosis, and demonstrates limited therapeutic efficacy in response to antibiotic treatment [1]. As a drug-resistant strain, Methicillin-resistant Staphylococcus aureus (MRSA) frequently induces ocular infections, thereby presenting significant challenges to the clinical management of endophthalmitis [41,42]. Thus, to evaluate the efficacy of C_6_PLL_10_-Gua in the treatment of endophthalmitis, an infectious endophthalmitis model was effectively developed in Sprague-Dawley rats via the administration of a MRSA solution. (Fig. 4a). The MRSA-infected rats were subsequently divided into four groups for *in vivo* analysis: a group receiving PBS eye drops, a group receiving intravitreal Van injections, a group treated with C_6_PLL_10_ eye drops, and a group treated with C_6_PLL_10_-Gua eye drops. As illustrated in Fig. 4b, on day 1, all groups exhibited corneal turbidity, anterior chamber fibrin exudation, conjunctival congestion, corneal edema, and substantial purulent secretions. Notably, on day 3, the C_6_PLL_10_-Gua group showed notable improvement with reduced fibrin exudation, less edema, and nearly normal pupil appearance, likely due to the rapid bactericidal effects of C_6_PLL_10_-Gua. The Van group showed a marked decrease in inflammation after 5 days due to its bacteria-clearing properties. Conversely, the PBS group had increased fibrin exudation, conjunctival congestion, and pupil adhesion due to bacterial growth and immune response. The C_6_PLL_10_ group had minimal therapeutic effects, less effective than C_6_PLL_10_-Gua. The antibacterial properties of C_6_PLL_10_ clear bacteria from the conjunctival capsule and prevent their entry into the eye, resulting in less inflammation compared to the PBS group. In the PBS group, bacterial growth triggers more severe inflammation by attracting neutrophils and macrophages. On 7 Day, the C_6_PLL_10_-Gua and Van groups exhibited clear eyes devoid of any signs of inflammation or infection. Conversely, the PBS group and the C_6_PLL_10_ group continued to display purulent secretion in the pupil area, with a minor amount of fibrin remaining unabsorbed. Furthermore, as seen from Fig. 4c the degree of ocular inflammation in each group was assessed based on the criteria established in previous literature. The control group exhibited a significant reduction in inflammation scores on day 7, likely attributable to the innate immune response effectively eliminating the infecting bacteria. In contrast, the eyes treated with C_6_PLL_10_-Gua exhibited a reduced inflammatory response during the treatment period compared to the other four groups. After clinical inflammation scoring, on days 1, 3, 5, and 7 of the treatment regimen, samples of aqueous humor and vitreous cavity fluid were collected daily from the rats, plated on LB agar, and incubated in a bacterial incubator for 24 h. As illustrated in Fig. 4d and e, the majority of the bacterial population was eradicated following treatment with C_6_PLL_10_-Gua. Over time, the number of bacterial colonies diminished significantly, with virtually no colonies observed by day 7, a result comparable to that observed in the Van group. In comparison with the PBS group, C_6_PLL_10_-Gua group demonstrated the ability to eliminate most bacteria within 3 days and effectively controlled bacterial proliferation over a 7-day period, the quantity of colonies decreased by three logarithms (*P* < 0.001).

Similar to MRSA, endophthalmitis induced by *E. faecalis* is also characterized by rapid progression and poor prognosis. Thus, the therapeutic efficacy of C_6_PLL_10_-Gua against *E. faecalis*-induced endophthalmitis was investigated. As depicted in Fig. S11, slit lamp photography and bacterial smear plate counting assessments demonstrated that C_6_PLL_10_-Gua exerted significant therapeutic effects on endophthalmitis caused by *E. faecalis*.

**Fig. 5:**
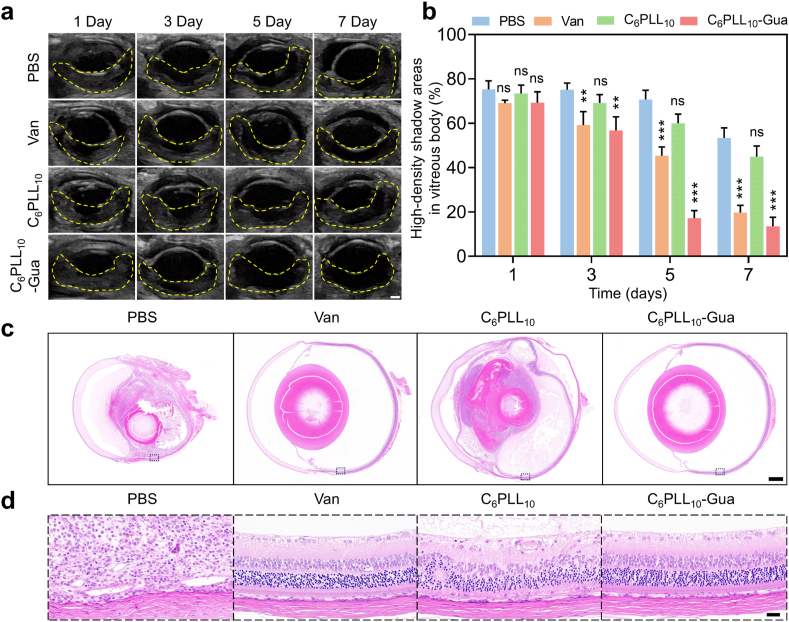
a) Ultrasound images representative of MRSA-infected rat eyes obtained at 1, 3, 5, and 7 days following various treatment interventions, yellow dotted line: vitreous cavity. Scale bar: 100 μm. b) Areas of high-density shadow in vitreous cavity. The high-density shadow rate of endophthalmitis on days 3, 5, 7 of C_6_PLL_10_-Gua group and van group was lower than that in PBS group, and the difference was statistically significant (∗∗*p* < 0.01, ∗∗∗*p* < 0.001). There was no significant difference between C_6_PLL_10_ group and PBS group (^ns^*p* > 0.05). c) The representative H&E images of eyeballs. Scale bar: 500 μm. d) The enlarged view of dashed rectangle region in picture c, the representative H&E images of retina. Scale bar: 25 μm. (For interpretation of the references to colour in this figure legend, the reader is referred to the Web version of this article.)

To further investigate the inflammation of the vitreous body in MRSA-induced endophthalmitis, ultrasound examinations were conducted on days 1, 3, 5, and 7. As illustrated in Fig. 5a and b, a high-density fibrin exudate was detected in the vitreous cavity on day 1. On days 3, 5, and 7, compared with the PBS group, the eyes treated with C_6_PLL_10_-Gua or Van exhibited reduced areas of high density, aligning with the observed symptoms on the ocular surface (*P* < 0.01,*P* < 0.001). In accordance with the findings from anterior segment examination and B-ultrasonography, the eyes of the rats were removed and analyzed with H&E staining on day 7. As seen from Fig. 5c and d, the PBS group and the C_6_PLL_10_ group exhibited varying degrees of ocular structural damage, including retinal detachment, ocular atrophy, and significant infiltration of inflammatory cells, ocular tissue damage and inadequate blood supply in the PBS group led to eyeball atrophy, significantly reducing the size of the eyeball. However, the ocular structures in the C_6_PLL_10_-Gua group remained intact, with no evidence of inflammatory cell infiltration, similar to those of Van group. The electroretinogram (ERG) in the rat model for the C_6_PLL_10_-Gua group on the day 7 have evaluated (Fig. S9). The results indicated no significant difference when compared to the ERG of healthy rats, demonstrating that the retinal function is preserved in the C_6_PLL_10_-Gua group. Immunohistochemical analysis of myeloperoxidase (MPO) was conducted in the rat model of endophthalmitis to further assess intraocular inflammation (Fig. S10). In comparison with the PBS treated group, MPO expression was substantially decreased in the C_6_PLL_10_-Gua group. Collectively, these findings indicate that C_6_PLL_10_-Gua demonstrates remarkable therapeutic efficacy in treatment of bacterial endophthalmitis without inducing significant inflammatory response of ocular tissues.

### 2.6 Biocompatibility study of C_6_PLL_10_-Gua

**Fig. 6:**
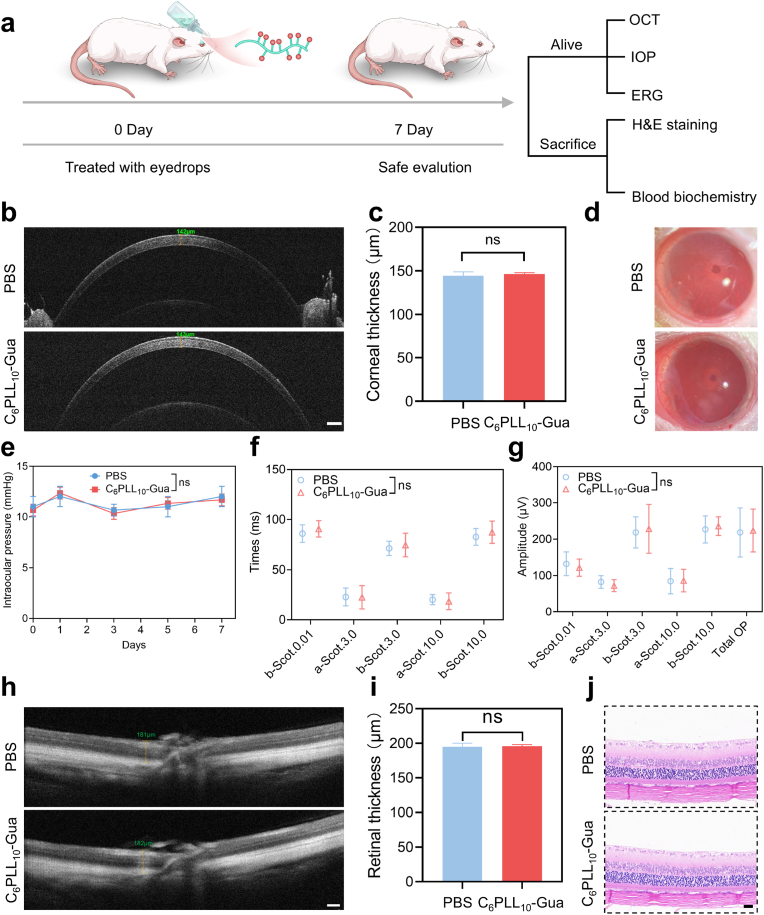
a) Ocular biosafety of C_6_PLL_10_-Gua. (b) Representative OCT b-scans of cornea after eye drops for 7 days straight respectively, PBS and C_6_PLL_10_-Gua. Scale bar: 200 μm. c) Statistical analysis of corneal thickness from OCT images. ^ns^*p* > 0.05. d) Typical photographs of rat eyes under slit lamp after eye drops for 7 days straight respectively, PBS and C_6_PLL_10_-Gua. e) Measurement of the IOP (n = 3 eyes per group). f) and g) Electrophysiological evaluations of retinal function in various groups. (n = 3 rat per group). h) Typical OCT b-scans of retina after eye drops for 7 days straight respectively, PBS and C_6_PLL_10_-Gua. Scale bar: 100 μm. i) Statistical analysis of retina thickness according to the OCT images. j) H&E images of retina after eye drops for 7 days straight respectively, PBS and C_6_PLL_10_-Gua. Scale bar: 20 μm.

To assess the biosafety of the C_6_PLL_10_-Gua *in vivo*, we then administered topical instillations of PBS or C_6_PLL_10_-Gua to uninfected wild-type rats. The physiological retina structure and function were assessed 7 d after instillation (Fig. 6a). The cornea was examined and imaged in cross-section utilizing an OCT system. By contrast with the PBS group, topical instillation of C_6_PLL_10_-Gua exhibited no impact on the structural integrity or thickness of the cornea (Fig. 6b and c). As shown in Fig. 6d and S12, in the eye slit-lamp photographs and corneal fluorescein sodium staining photographs, there were no obvious inflammatory symptoms and corneal epithelial injury on ocular surface. All the above results suggesting that the topical instillations of C_6_PLL_10_-Gua was safe for the cornea. The intraocular pressure (IOP) monitoring after the instillation of C_6_PLL_10_-Gua revealed no significant alterations throughout the experiment when compared with PBS group (Fig. 6e). Subsequently, retinal function was evaluated through the application of electroretinography (ERG), which proved no effect of topical instillation of C_6_PLL_10_-Gua on the normal electrophysiological activity of the retina (Fig. 6f and g). Furthermore, we determined the retinal thickness based on corneal images obtained through the OCT system (Fig. 6h and i), which proved no adverse effect on retina thickness. After euthanasia, the structural integrity of the retinal layers in the rats was evaluated. H&E staining of retinal sections was conducted, revealing that the C_6_PLL_10_-Gua group exhibited no significant alterations over time in terms of retinal inflammation or structural phenotype. (Fig. 6j). To further assess *in vivo* biocompatibility, PBS and C_6_PLL_10_-Gua were administered to the eyes of rats without bacterial infection for a duration of 7 days, after which blood biochemical indices were evaluated. As illustrated in Fig. S13, the indices for rats treated with C_6_PLL_10_-Gua were largely comparable to PBS group. Additionally, H&E staining of main organs indicated an absence of tissue disarrangement or inflammation (Fig. S14). The above findings collectively suggest that C_6_PLL_10_-Gua exhibits a high degree of biocompatibility *in vivo*. The cationic compound C_6_PLL_10_-Gua demonstrates a capacity to effectively disrupt bacterial membranes through electrostatic interactions. Conversely, mammalian cell membranes, exhibit relatively weak electrostatic interaction with the cationic C_6_PLL_10_-Gua, thereby resulting in reduced cytotoxicity.

## 3 Conclusion

In summary, we successfully prepared a special ocular barrier penetrating and antibacterial poly-(L-homoarginine) via a combination of ring-ROP of NCA monomers and post-polymerization modification. The obtained C_6_PLL_n_-Gua, demonstrated significant *in vitro* antibacterial properties. Notably, C_6_PLL_10_-Gua exhibited lower hemolytic toxicity and higher selectivity index to the bacteria compared with C_6_PLL_30_-Gua. Investigations into the antibacterial mechanisms revealed that C_6_PLL_10_-Gua effectively destroyed the integrity of bacteria membranes, resulting in bacteria death. Comparison with clinical antibiotics, C_6_PLL_10_-Gua exhibit rapid bactericidal kinetics and less likely to induce bacteria resistance. Notably, due to its distinct structural attributes, C_6_PLL_10_-Gua exhibits superior penetration capabilities in both *in vivo* and ex vivo tissues. This formulation of C_6_PLL_10_-Gua eye drops effectively traverses the ocular barrier, facilitating access to the posterior segment of the eye. Upon administration into the conjunctival sac, C_6_PLL_10_-Gua achieves rapid (<30 min) and sustained (>24 h) distribution in the retina, peaking at 6 h, via a non-corneal pathway. Finally, C_6_PLL_10_-Gua showed significant therapeutic effect in a MRSA induced rat endophthalmitis model with no significant side effects. Taken together, this study offers a secure and promising therapeutic approach for the treatment of clinical endophthalmitis infections.

## CRediT authorship contribution statement

**Ting Hua:** Writing – review & editing, Investigation, Data curation. **Tianzi Zhang:** Software. **Yi Tang:** Validation, Software. **Shuo Wang:** Software, Methodology. **Guowenlie Gao:** Validation, Software. **Chunsheng Xiao:** Writing – review & editing. **Pengqi Wan:** Writing – review & editing, Funding acquisition. **Hong Wu:** Writing – review & editing, Supervision, Resources, Project administration.

## Declaration of competing interest

The authors declare that they have no competing financial interests or personal relationships that could have appeared to influence the work reported in this paper. The authors declare no conflict of interest.

## Data Availability

All relevant data are available with the article and its Supplementary Information files, or available from the corresponding authors upon reasonable request.
