# Supplementary material for: Poly-(L-homoarginine) for the non-invasive treatment of endophthalmitis
Source: Mater Today Bio. 2025 Jun 2;33:101931. doi: 10.1016/j.mtbio.2025.101931 (PMC12173625; doi:10.1016/j.mtbio.2025.101931)
Supplement: Multimedia component 1 [file mmc1.docx]

**Poly-(L-homoarginine) for the non-invasive treatment of endophthalmitis**

Ting Hua^1,2^, [Tianzi Zhang](https://pubmed.ncbi.nlm.nih.gov/?term=Zhang+TZ&cauthor_id=29864918)^2^, Yi Tang^1^, Shuo Wang^1^, Guowenlie Gao^3^, Chunsheng Xiao^3^, Pengqi Wan^3*^ and Hong Wu^1*^

^1^Department of Ophthalmology, The Second Hospital of Jilin University, Changchun 130041, China.

^2^Affiliated Hospital of Inner Mongolia University for the Nationalities, Inner Mongolia, China.

^3^Key Laboratory of Polymer Ecomaterials, Changchun Institute of Applied Chemistry, Chinese Academy of Sciences. Changchun 130022, P. R. China

^*^ Corresponding authors/ To whom correspondence should be addressed. Prof. Hong Wu, Email: [wu_hong@jlu.edu.cn](mailto:wu_hong@jlu.edu.cn); Dr. Pengqi Wan, Email: wpengqi@ciac.ac.cn

**1. Experimental Section**

***1.1 Materials and animal***

*N^ε^*-Carbobenzoxy-L-lysine (L-Lys), 3,3'-Dipropylthiadicarbocyanine Iodide (DiSC3(5)) and 1% (*v/v*) crystal violet solution were purchased from Aladdin Biochemical Technology Co., Ltd. (Shanghai, China); Triphosgene was purchased from Darui finechem Co., Ltd. (Shanghai, China); Hydrogen bromide (33 wt.% in acetic acid) and trifluoroacetic acid (TFA) were purchased from Energy Chemical Co., Ltd. (Shanghai, China); Vancomycin (Van), levofloxacin and methicillin were sourced from Shanghai yuanye Bio-Technology Co., Ltd (Shanghai, China); *N*, *N*-dimethylformamide (DMF) and tetrahydrofuran (THF) were acquired from Sinopharm Chemical Reagent Co., Ltd. and subsequently depurated by a solvent purification system (MB SPS-800, MBRAUN, Germany); deionized water was refined by the Milli-Q system (Millipore Co., Billerica, MA, USA); Live and dead dye kit were procured from Sigma-Aldrich (Shanghai, China); *S. aureus* (ATCC 25923), MRSA (ATCC 43300), *E. coli*, ATCC 25922), *Pseudomonas aeruginosa* (*P. aeruginosa*, ATCC 15442), *Klebsiella pneumoniae* (*K. pneumoniae*, ATCC 700603), *Acinetobacter baumannii* (*A. baumannii*, ATCC 17904), *Enterococcus faecalis* (*E. faecalis,* ATCC 29212) were obtained from Luwei Technology (Shanghai, China). *Staphylococcus wardii* (*S. wardii*), and *Staphylococcus epidermidis* (*S. epidermidis*) were isolated from the conjunctival sac of clinical cataract patients. Prior to use, the samples used in the bacterial culture were autoclaved at 120 °C for 20 min.

The ^1^H NMR spectra were obtained using a Bruker AV-500 NMR spectrometer in deuterated water (D_2_O), or deuterated chloroform (CDCl_3_). The synthesized polymers' molecular weights and polydispersity (*Ɖ*) were measured using gel permeation chromatography (GPC) with a Waters 515 HPLC pump and a Waters 2414 Refractive Index Detector. Measurements via transmission electron microscopy were executed using a HITACHI HT7700 microscope from Tokyo, Japan, with an 80 kV accelerating voltage. Absorbance spectra were recorded using a Lambda 365 UV-Vis spectrophotometer from PerkinElmer, USA. Confocal laser scanning microscopy (CLSM) (Carl Zeiss, LSM 780, Germany) was used to observe the fluorescence of the samples. The intraocular pressure (IOP) was measured using a hand-held tonometer (Icare, Fenland). Electroretinograms (ERGs) were recorded and exported utilizing an RETI-Port device (IRC China). Optical coherence tomography (OCT) images were acquired with the Spectralis OCT system (Optovue, USA).

The female C57/BL/6 mice (6–8 weeks old) were purchased from Beijing Vital River Laboratory Animal Technology Co., Ltd. (Beijing, China). The female Sprague-Dawley (SD) rats (6–8 weeks old) were purchased from Liaoning Changsheng biotechnology co., Ltd. (Liao Ning, China). All of the animals received care according to Guide for Care and Use of Laboratory Animals. All operations were approved by Animal Care and Use Committee at Jilin University, and complied with all relevant ethical regulations.

***1.2 C_x_-PLL_n_ synthesis and Characterization***

The synthesis of ε-benzyloxycarbonyl-_L_-lysine *N*-carboxyanhydride (ZLL-NCA) was conducted following a method previously documented in the literature.^1^ C_6_-PLL_10_ was synthesized through the ring-opening polymerization (ROP) of ZLL-NCA in accordance with a previous reference. ^2^ The representative scheme was shown in Figure S1. Initially, 0.34 g of *N*-hexylamine (3.26 mmol) was solvated in 30 mL of anhydrous DMF. Subsequently, 10.0 g of ZLL-NCA (32.6 mmol) was solvated in 120 mL of anhydrous DMF and immediately puted into the *N*-hexylamine solution. The resulting mixture was stirred under a nitrogen atmosphere at ambient temperature for 72 h. Following this, the solution was treated with saturated brine. Upon drying under vacuum, C_6_-PZLL_10_ was isolated as a white solid, with a yield of 64%.

Next, 4 g of C_6_-PZLL_13_ (3.36 mmol) was dissolved in 35 mL of trifluoroacetic acid (TFA). Then, fourteen milliliters of hydrogen bromide (33 wt.% in acetic acid) were added to the reaction mixture. The solution was then stirred at room temperature for two hours. Subsequently, dialysis was performed against deionized water for a duration of two days using a dialysis bag with a molecular weight cut-off (MWCO) of 500 Da. Following lyophilization, the C_6_-PLL_10_ compound was isolated as a white solid, with a yield of 58%. The synthesis of C_6_-PLL_30_ was conducted following the same protocol.

***1.3 C_6_PLL_n_-Gua synthesis***

C_6_PLL_10_-Gua was synthesized through a post polymerization guanylation method was used.^1^ First, 2 equiv of PCH and 2 equiv of potassium carbonate relative to the amine groups were added to the aqueous solution of C_6_-PLL_10_. Following a 12 h stirring period at 55 °C, the solution underwent dialysis against deionized water for a duration of two days using a dialysis bag with a molecular weight cut-off (MWCO) of 500 Da. Subsequent to lyophilization, the compound C_6_-PLL_30_-Gua was isolated as a white solid, achieving a yield of 76%. Synthesis of the C_6_-PLL_30_-Gua was employed according to the protocol above.

***1.4*** ***Minimum Inhibitory Concentration (MIC) Measurements***

*S. aureus, E. coli*, MRSA*, P. aeruginosa, K. pneumoniae, A. baumannii, S. wardii,* *S. epidermidis* and *E. faecalis* were incubated in MH medium at 37℃. For the determination of the MIC, MH solutions of C_6_PLL_10_-Gua at various concentrations were prepared. Subsequently, a volume of 2 μL of bacterial suspension, containing 1 × 10^8^ CFUs, was introduced into 200 μL of the culture medium within a microplate. Following a brief agitation of the plate, the absorbance value at time zero was measured at a wavelength of 600 nm utilizing a Spark Multimode microplate reader (Tecan, Switzerland). Subsequent to incubation of the plate at 37°C for a duration of 24 h, the optical density of the microbial solution was assessed at a wavelength of 600 nm. The MIC was determined as the lowest concentration at which no visible bacterial growth was detected.

***1.5 Hemolysis Assay***


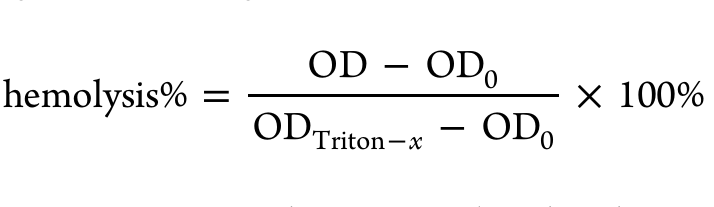
A suspension containing 4% rabbit erythrocytes was sourced from Yuan Mu Biological Technology Co. Ltd., located in Shanghai, China. After spinning at 2000 rpm for 5 minutes, the erythrocytes were harvested and then resuspended in PBS at pH 7.4. Subsequently, A volume of 400 μL of the erythrocyte suspension (4% in PBS, pH 7.4) was incubated with a range of concentrations of C_6_PLL_n_-Gua (400 μL, dissolved in PBS, pH 7.4) for a duration of 2 h. PBS buffer (PBS, 400 μL, pH 7.4) and Triton X (1% v/v, 400 μL) functioned as the negative and positive controls. Subsequently, the supernatant isolated via centrifugation was transferred to a microplate. Hemolysis was assessed and the hemolysis rate was analyzed by a specific formula:

The absorbance values at 576 nm for the positive and negative control wells are represented by OD_Triton−x_ and OD_0_ respectively. The absorbance value for the wells treated with C_6_PLL_n_-Gua is denoted as OD, and is similarly measured at 576 nm.^3^

***1.6 In vitro killing efficiency and kinetics.***

The time-kill kinetics of C_6_PLL_10_-Gua against bacteria were evaluated. In brief, bacterial suspensions were prepared in phosphate-buffered saline (PBS) to achieve a final concentration of 1 × 10^6^ colony-forming units per milliliter (CFU/mL). The bacterial suspensions were subsequently treated with C_6_PLL_10_-Gua or antibiotic at concentrations equivalent to 1 ×, 2 ×, and 4 × the minimum inhibitory concentration (MIC) for durations of various time points. At every designated time interval, the number of bacteria was measured using the plate counting technique. PBS group served as the negative control.

***1.7*** ***Live/Dead Bacterial Staining Assay***

A suspension of *S. aureus* (1 mL, 10^6^ CFU/mL) was exposed to 1 mL of C_6_PLL_10_-Gua aqueous suspension at a concentration of 2 times the MIC and incubated with agitation at 37 °C for half-hour. Following incubation, the bacterial cells were subjected to centrifugation and subsequently stained with SYTO 9 and propidium iodide (PI) for half-hour in darkness. The stained mixture underwent centrifugation and was rinsed twice with PBS. The processed bacterial suspension was then placed a glass slide and secured with coverslips for stabilization. Finally, the samples were analyzed using a LSM510 confocal microscope (Nikon 108, Japan). The identical experimental protocol was implemented for *E. coli*.^2^

***1.8 Morphology Change of Bacteria Cells Imaged by Scanning Electron Microscopy (SEM) and Transmission electron microscope (TEM)***

To investigate the impact of C_6_PLL_10_-Gua on bacterial morphology, bacterial cells cultured in Mueller-Hinton (MH) broth were incubated with or without C_6_PLL_10_-Gua, following a protocol analogous to that employed for minimum inhibitory concentration (MIC) determinations. Specifically, bacterial suspensions at a concentration of 1 × 10^6^ CFU/mL were treated with C_6_PLL_10_-Gua at a concentration equivalent to twice the MIC for a duration of half-hour. Subsequently, the bacteria were harvested, subjected to centrifugation at 5000 rpm for 5 min, and washed twice with PBS. Subsequently, bacterial cells were fixed in a 4% paraformaldehyde solution for 1 h, after rinsing with DI water. The samples underwent dehydration through sequential exposure to ethanol solutions at gradient concentration, then obseved by SEM. The morphological alterations in bacteria subjected to C_6_PLL_10_-Gua treatment were further examined using TEM. The treatment protocol was consistent with the aforementioned procedures, with bacterial samples obtained from the copper web analyzed through TEM.

***1.9 Prevention of Resistance Development.***

The study utilized *S. aureus* to investigate the potential for bacterial resistance development against C_6_PLL_10_-Gua, with methicillin serving as a control. MICs for both C_6_PLL_10_-Gua and methicillin were initially determined following established protocols, with these initial MIC values designated as MIC_0_. Subsequently, MIC_1_ was assessed after exposing the bacteria to each respective antibacterial agent at a concentration of 0.5 × MIC for 24 h, followed by MIC determination. This procedure was conducted over 14 bacterial generations to assess the evolution of drug resistance.

***1.10 Evaluation of DNA, ATP Leakage***

The concentrations of DNA and ATP released by bacteria following various treatments were quantified. The optical density (OD) at 260 nm was assessed using a UV-VIS spectrophotometer to quantify the DNA released. ATP concentrations were measured utilizing an ATP assay kit (MAK190-1KT, Sigma-Aldrich). Fluorescence intensity was evaluated using an enzyme-linked fluorescence assay.

***1.11 Membrane permeability of bacteria***

PI is a red-fluorescent stain that binds to DNA and RNA,^4^ increasing its fluorescence 20- to 30-fold. As a membrane-impermeant stain, PI only labels bacteria with compromised inner membranes, making it useful for assessing changes in membrane permeability after antibacterial treatment. To assess bacterial inner membrane permeability, add 5 μL of 1 mM PI to 1 mL of resuspended *S. aureus* or *E. coli* cells. Incubate in the dark for 30 min at room temperature. Set the spectrometer to PMT voltage: 700 V, excitation: 535 nm, emission: 617 nm. Clean the fluorescence cuvette with water and ethanol. Add 1 mL of the culture to the cuvette, insert it into the spectrometer, and begin recording data.

***1.12 In vivo biosafety test***

1.12.1 Intraocular Pressure (IOP) Measurement

The IOP measurement assessed using a hand-held tonometer (Icare, Fenland). Initial IOP measurements were obtained for all rat, which were subsequently randomized into two groups: PBS and C_6_PLL_10_-Gua. Following administration of 10 μL of either C_6_PLL_10_-Gua or PBS into the rats’ eyes twice daily, IOP changes were monitored on the 1st, 3rd, and 7th days.

1.12.2 Electroretinogram (ERG)

Scotopic ERGs were conducted following the International Society for Clinical Electrophysiology of Vision's protocol. On the 7th day post-treatment, rats were dark-adapted for 12 h. Before recording various ERG responses, the rats were anesthetized, experienced pupil dilation, placed on a 37°C heating pad. Each eye was administered a 2.5% methylcellulose gel, and annular gold electrodes were placed on the corneas. Reference and ground electrodes were positioned on the mid-frontal head and tail areas. Rat groups were exposed to varying light intensities: −2.5, −0.5, −0.02, and 0.5 log (cd s m^−2^). The a-wave and b-wave amplitudes and implicit times were recorded using an RETI-Port device (IRC CHINA).

1.12.3 Optical Coherence Tomography (OCT)

After the above treatment, at the 7th day, OCT imaging was used to track changes in corneal and retinal thickness. Spectralis OCT was used to obtain the images (Optovue, the United States).

1.12.4 Fluorescein Sodium (FLS) Staining Test

10 μL of C_6_PLL_10_-Gua or PBS was dropped onto the eyes of rats twice daily for seven consecutive days. Then, a 1% FLS (w/v) solution was instilled into the conjunctival sac of the lower eyelid in rats. Finally, the corneal epithelium was examined using the cobalt blue light of a slit lamp microscope after a 60 s interval.

1.12.5 Serum Chemistry Analysis and Histology

For biosafety evaluation, serum was collected for blood biochemical analysis after treatment. The rat's eyes and major organs were fixed in 10% neutral-buffered formalin, embedded in paraffin, sectioned at 6 µm, and stained with H&E. Images were captured using an Olympus microscope, with data from at least three independent experiments.

***1.13 Distribution of C_6_PLL_10_-Gua -FITC in vivo and in vitro***

1.13.1 Transport efficiency across *in vitro* ARPE-19 cell monolayer

The *in vitro* ARPE-19 cell monolayer model was developed following previously described protocols, with minor modifications.^5^ In summary, ARPE-19 cells were seeded onto the apical surface of rat tail collagen type I-coated polyester membrane filters (6.5 mm diameter, 0.4 μm pore size, Corning) at a density of 5,000 cells per well. The integrity of the barrier was assessed by measuring transepithelial electrical resistance (TEER) using an epithelial volt-ohm meter (Millipore, USA). Monolayers exhibiting a transepithelial electrical resistance (TEER) exceeding 100 Ω cm², indicative of the successful formation of an intact ARPE-19 monolayer suitable for *in vitro* simulation of the retinal epithelium, were selected for subsequent experiments. The apical surface of these monolayers was exposed to either C_6_PLL_10_-Gua-FITC, C_6_PLL_10_-FITC, or left untreated, in 100 μL of D-Hanks balanced salt solution (D-HBSS, pH=7.2), while the basolateral side was maintained in 600 μL of D-HBSS. At 30 min intervals, a 200 μL aliquot was extracted from the basolateral compartment and replaced with an equivalent volume of fresh D-HBSS. The fluorescence intensity of the extracted sample was quantified using a microplate reader. Additionally, monolayers that had undergone transportation were examined using a confocal laser scanning microscope.

1.13.2 Cumulative permeability of C_6_PLL_10_-Gua -FITC using Franz diffusion

The corneas or posterior segments of New Zealand rabbits were dissected and positioned between the donor and receptor chambers of a Franz diffusion cell. After ensuring no leakage, each cell was placed on a thermostatic stirrer. Various samples (1 mL) were added to the donor chamber, and PBS (7 mL) was added to the receptor chamber. A 0.3 mL receptor solution was collected and replaced with fresh solution at 0.5, 1.5, 2, 2.5, 3, 3.5, and 4 h. FITC concentration was measured via fluorescence, and cumulative permeability was calculated using a specific formula.

Cumulative permeability = [*R_n_* × *V*_1_ + (*R_n_*_−1_ + *R_n_*_−2_ + *R_n_*_−3_ … + *R*_1_) × *V*_2_] × 100%/*R*_all_ × *V*_3_

Let *R_n_* be the fluorescence of the receptor solution at time n, *R*_n−1_ at time *n*−1, and *R*_1_ at time 1. Rall is the fluorescence of the donor chamber sample. *V*_1_ is the total receptor solution volume (7 mL), *V*_2_ is the volume of each collected solution (0.3 mL), and *V*_3_ is the donor chamber sample volume (1 mL).

1.13.3 Distribution *in vivo*

10 μL of C_6_PLL_10_-Gua-FITC was applied to mice's eyes twice daily. Mice were euthanized at 0.5, 1, 6, 12, 18, and 24 h, and their eyes were fixed in 4% PFA for 24 h. The eyes were then soaked in 10% sucrose for 1 hour, 20% sucrose for 2 h, and 30% sucrose overnight. Frozen sections were made using OCT and treated with 200 μL of DAPI anti-fade. They were examined under an inverted fluorescence microscope, and tissue distribution images were analyzed for fluorescence intensity using confocal microscope software.

***1.14 Development of endophthalmitis model***

To assess the therapeutic effect of C_6_PLL_10_-Gua on endophthalmitis, 24 female Sprague-Dawley (SD) rats (approximately 180 g each) were used to create a bacterial endophthalmitis model. The rats were divided into four groups: control, vancomycin (Van), C_6_PLL_10_-Gua, and C_6_PLL_10_. Prior to experiments, rats were anesthetized with 3.5% pentobarbital sodium (50 mg/kg) via intraperitoneal injection, and their eyes were numbed with 0.5% alcaine and dilated with tropicamide drops. To prevent high intraocular pressure, a 30-gauge syringe needle released a small amount of aqueous humor. A Hamilton syringe with a 30-gauge needle then injected 10 μL of MRSA solution (200 CFU) into the rat's vitreous. The needle was inserted vertically from the limbus, and after injection, it remained in place for a short time with slight shaking. Following the successful establishment of a MRSA endophthalmitis model, 10 μL PBS (control group), 10 μL C_6_PLL_10_-Gua (C_6_PLL_10_-Gua group) or 10 μL C_6_PLL_10_ (C_6_PLL_10_ group), were dropped in the eyes of rat twice one day, and 10 μL vancomycin (Van group) were injected into vitreous cavity to evaluate the therapeutic effect, with eye surface inflammation monitored via slit lamp images and vitreous humor were collected at 1, 3, 5, and 7 days. Eyeballs were quickly frozen on dry ice. The cornea was cut along the limbus to collect aqueous humor, then the lens was removed to obtain vitreous humor, which was pooled from three rats per group and placed on ice. Before cryogenic pulverization, vitreous humor was mixed with 2.5 μL/mg of PBS. The sample was centrifuged at 13,000 rpm for 10 min at 4 °C. The supernatant was frozen at −80 °C, and the sediment was resuspended in PBS and diluted 1000-fold for bacterial culture on an agar plate. To further investigate intraocular inflammation, B-ultrasound images of anesthetized rat eyeballs were captured using a GE LOGIQ E8 small animal imaging system.

The *E. faecalis* rat endophthalmitis model was established by the same method as the MRSA rat endophthalmitis model. 8 female SD rats (approximately 180 g each) were used to create a bacterial endophthalmitis model. The rats were divided into two groups: control and C_6_PLL_10_-Gua treated group. Following the successful establishment of the *E. faecalis* endophthalmitis model, 10 μL PBS or 10 μL C_6_PLL_10_-Gua were dropped onto the eyes of rat twice one day, with eye surface inflammation monitored via slit lamp images at 1, 3, 5, and 7 days. Following the collection of the eyeballs, bacterial cultures were prepared on agar plates employing the previously described methodology.

***1.15 Histopathological study***

On the seventh day, the excised ocular tissues were fixed in a 4% paraformaldehyde solution for 12 h and subsequently embedded in paraffin to facilitate the preparation of tissue sections with a thickness of 5 μm. These specimens were then subjected to hematoxylin and eosin (H&E) staining and examined using an optical microscope.^6^

***1.16 Immunohistochemical staining***

Immunohistochemical staining for myeloperoxidase (MPO), the predominant enzyme located within the azurophilic granules of neutrophils, was utilized to investigate the extravasation of neutrophils to inflammatory sites. This analysis was conducted on paraffin-embedded sections of vitreous body tissue following treatment. Images were acquired using an optical microscope.

***1.17 Statistical analysis***

All experimental data are presented as mean ± standard deviation. Statistical analyses were conducted using Student's t-test, with differences deemed statistically significant at **p*< 0.05, ***p* < 0.01, and ****p*< 0.001.


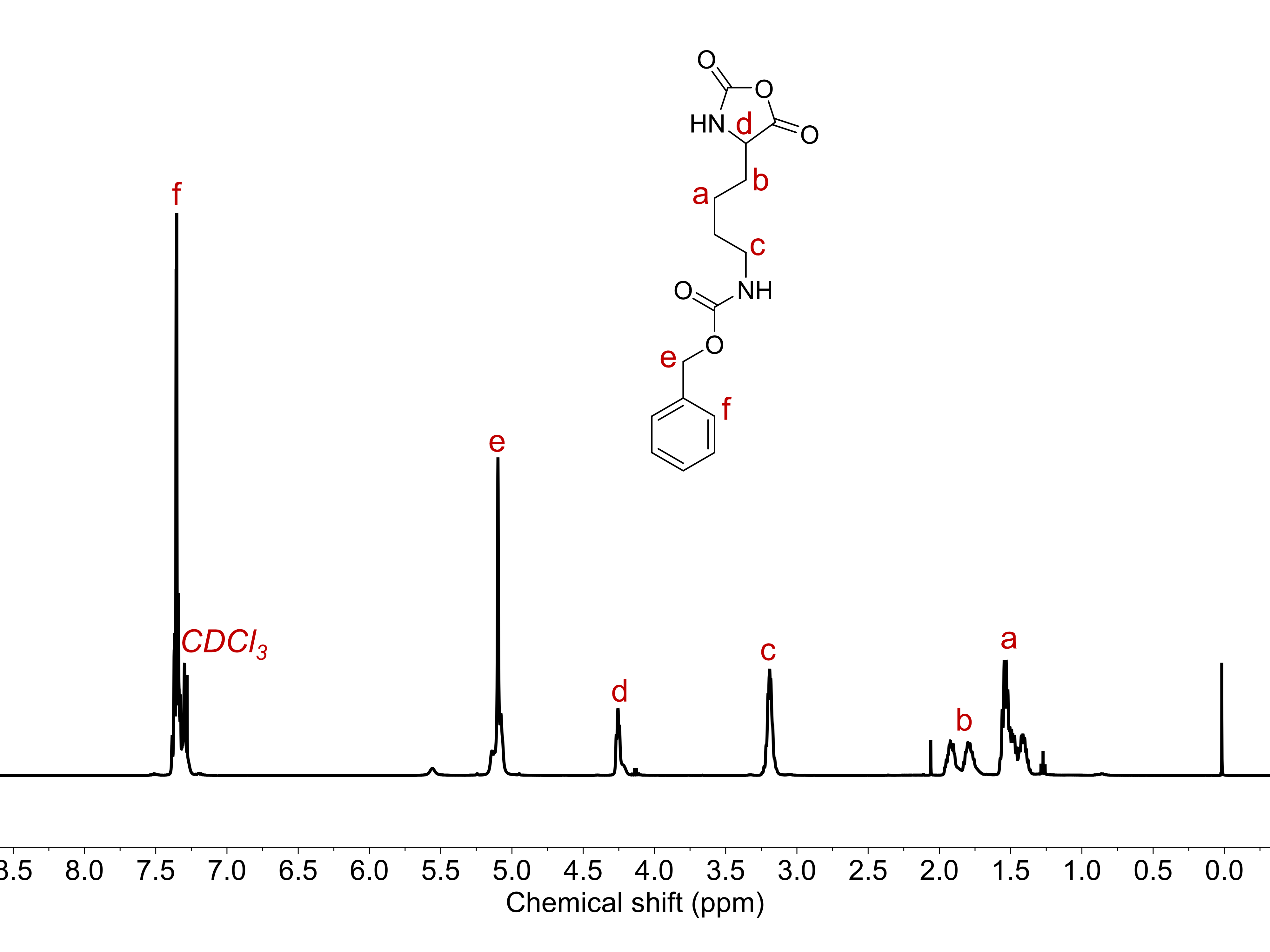
**Figure S1.** Typical ^1^H NMR spectrum of ZLL NCA in CDCl_3_.


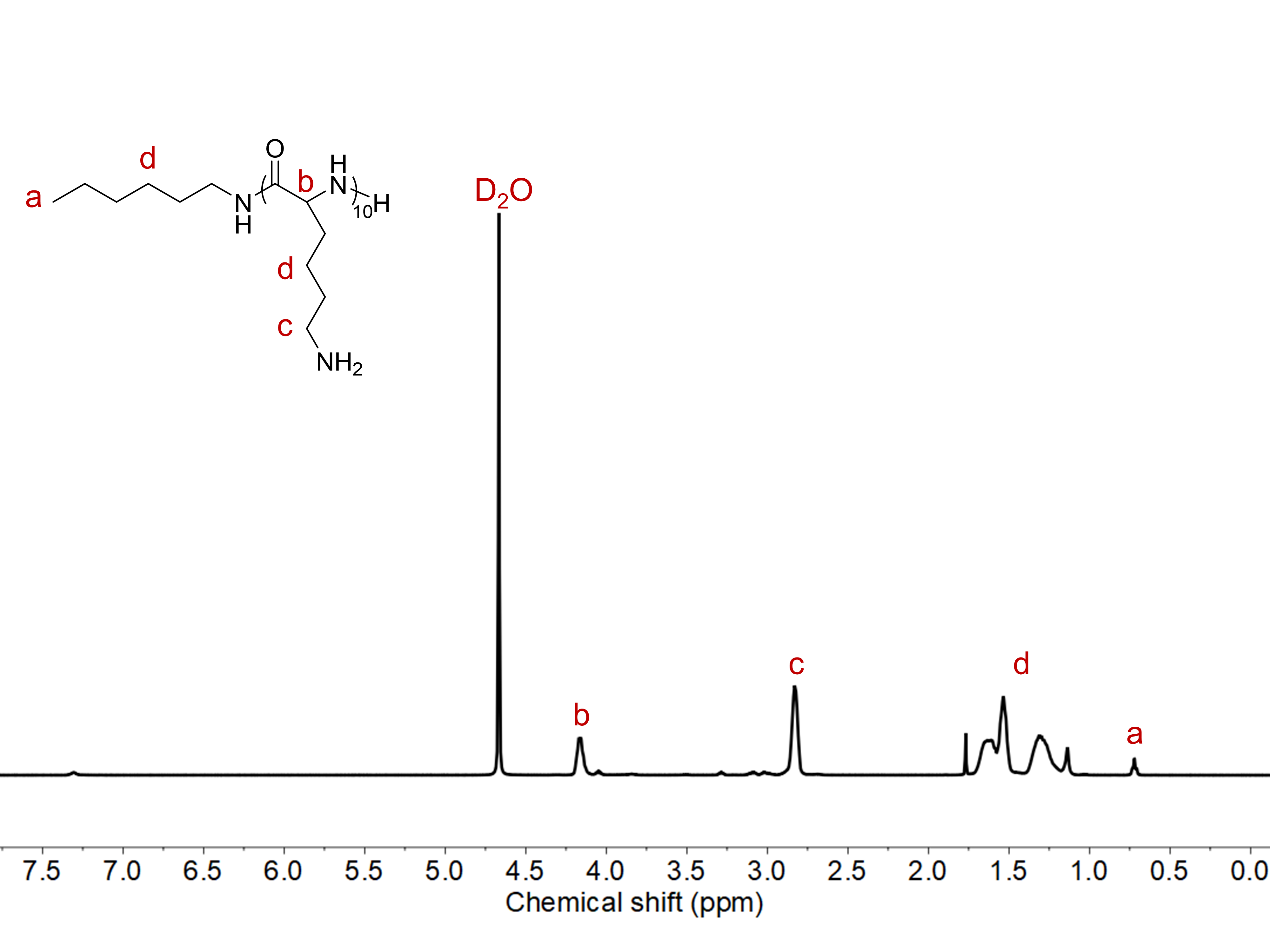


**Figure S2.** Typical ^1^H NMR spectrum of C_6_PLL_10_ in D_2_O.


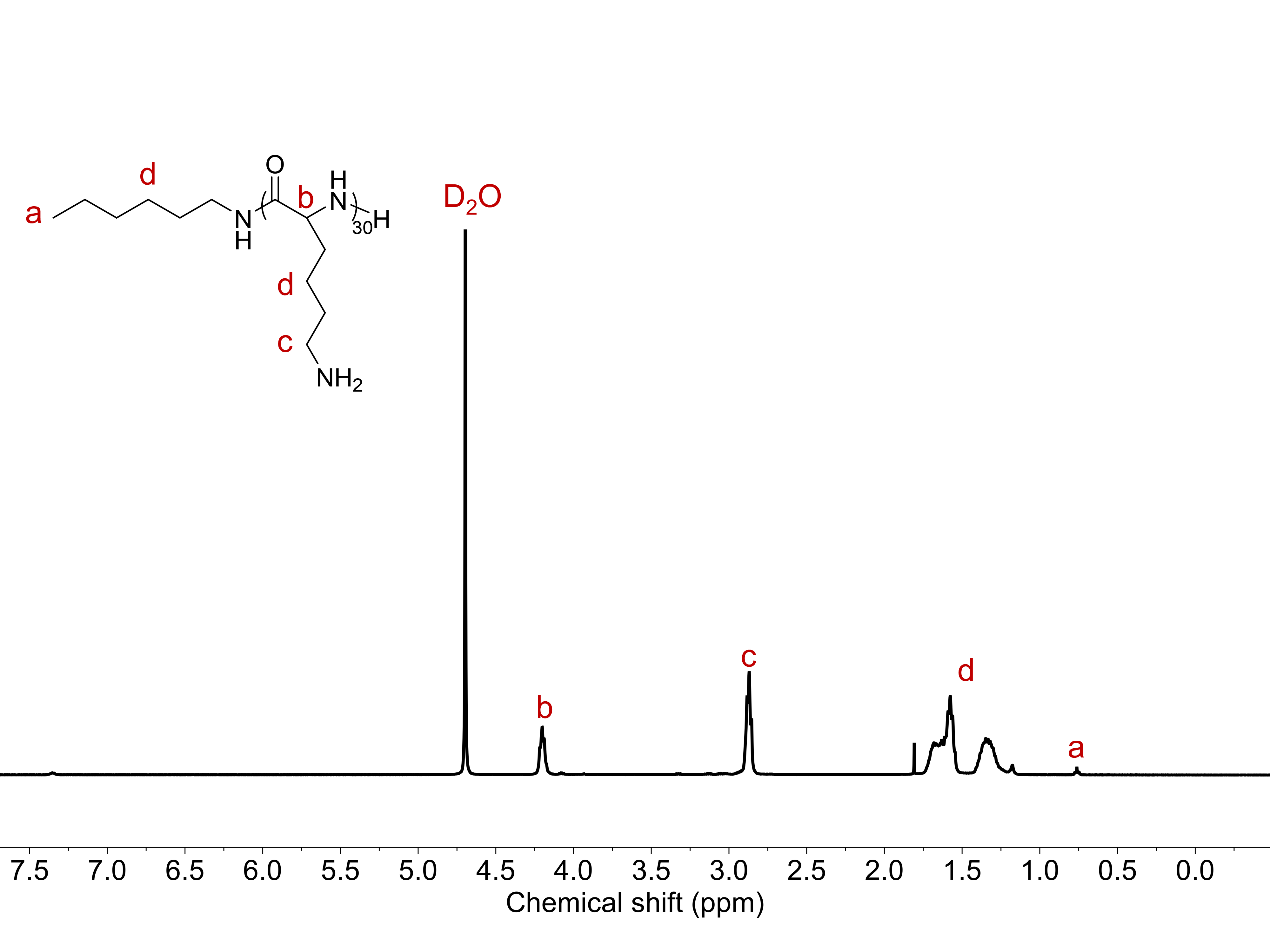
 **Figure S3.** Typical ^1^H NMR spectrum of C_6_PLL_30_ in D_2_O.


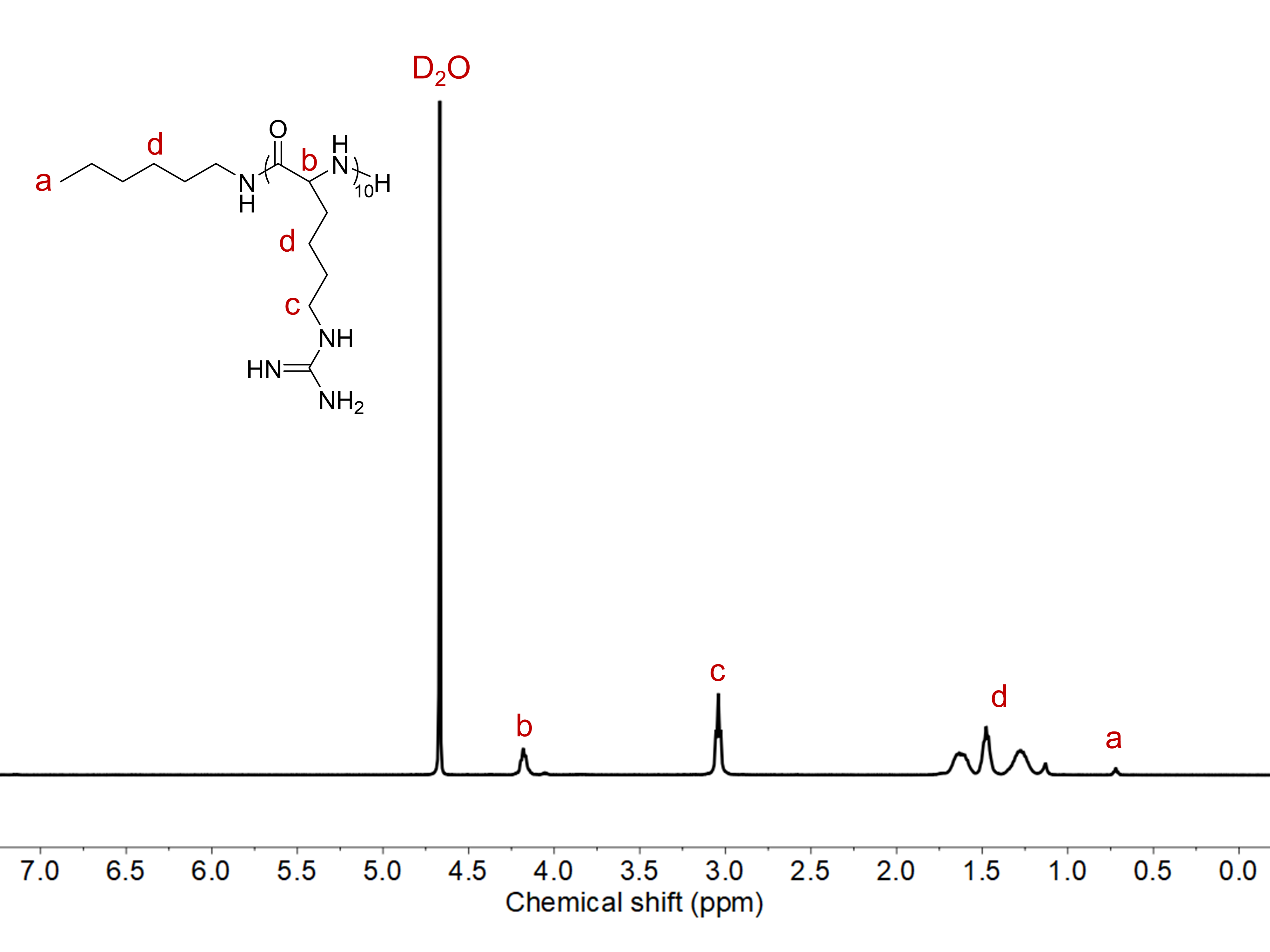
 **Figure S4.** Typical ^1^H NMR spectrum of C_6_PLL_10_-Gua in D_2_O.


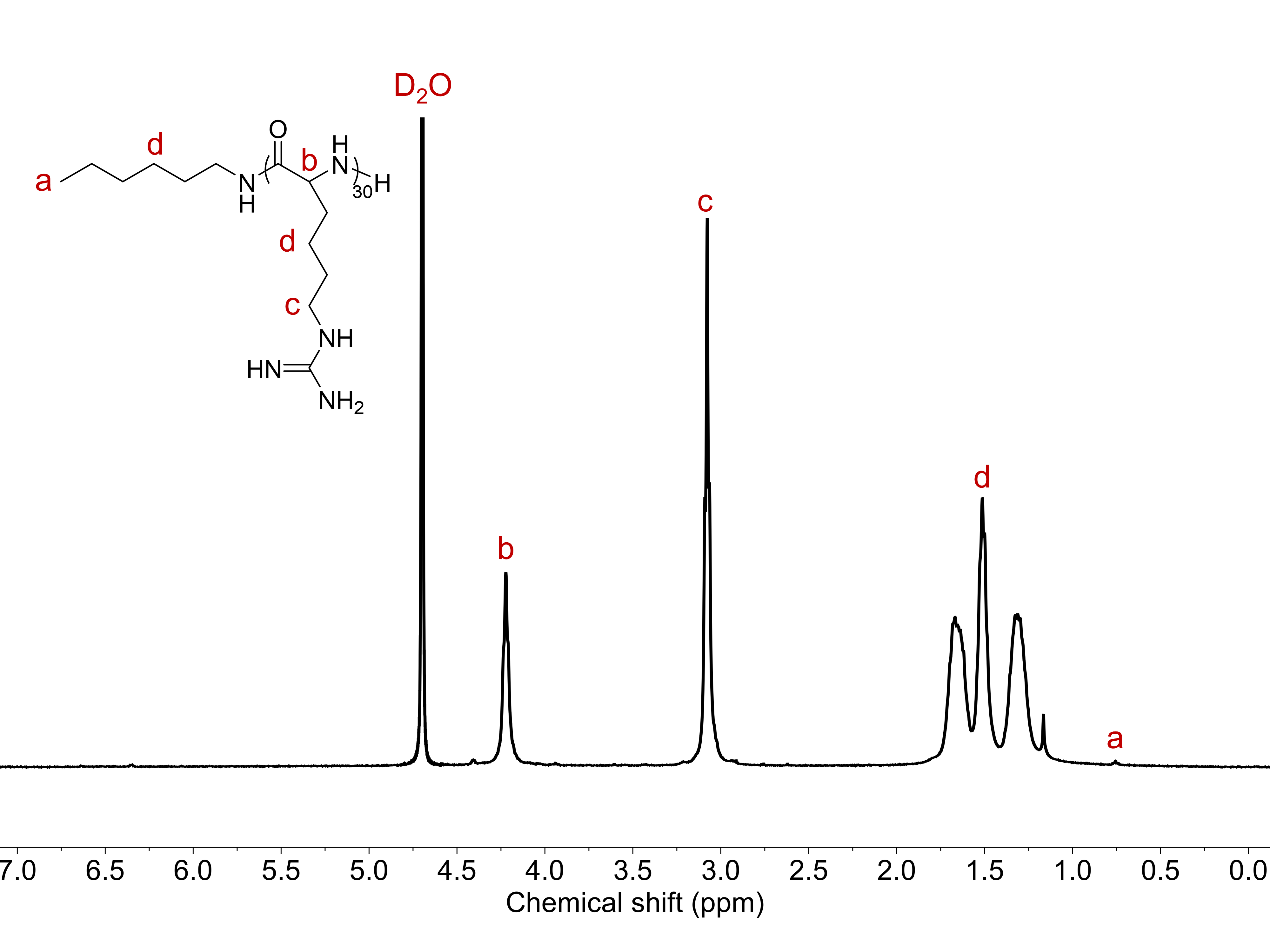
 **Figure S5.** Typical ^1^H NMR spectrum of C_6_PLL_30_-Gua in D_2_O.


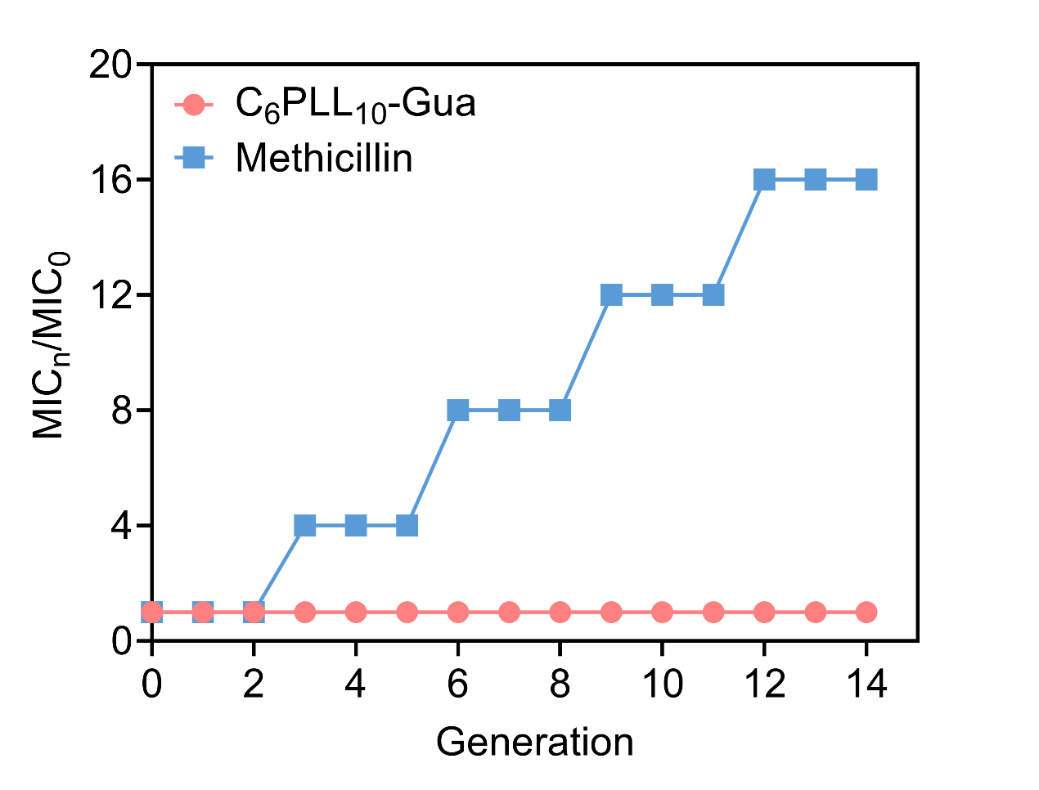


**Figure S6.** Resistance of *S. aureus* to C_6_PLL_10_-Gua and methicillin at subtherapeutic concentrations (0.5 × MICn) after 14 generations.


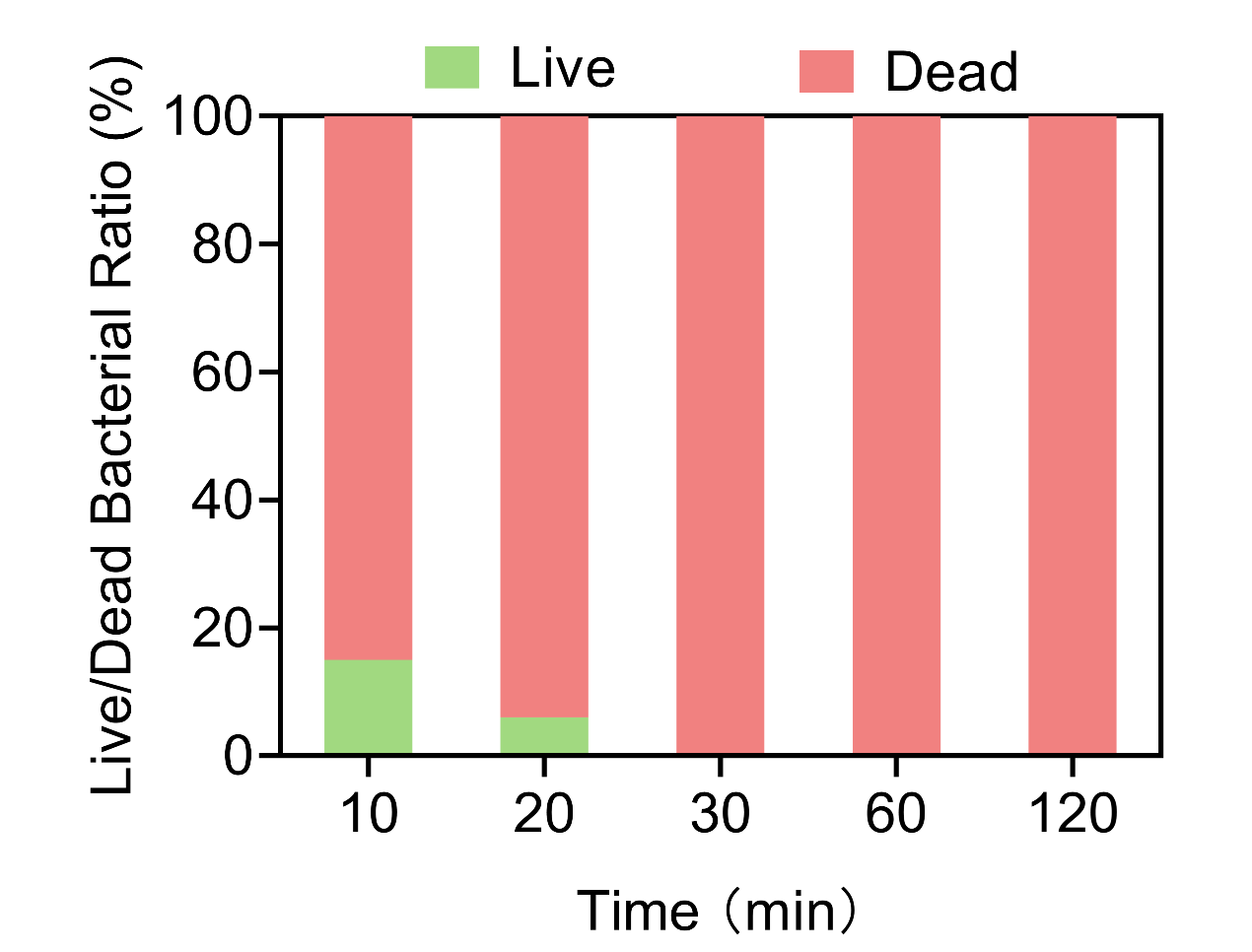
**Figure S7.** Statistical diagram of semi-quantitative fluorescence analysis of *S. aureus* treated with 2 × MIC of C_6_PLL_10_-Gua for different.


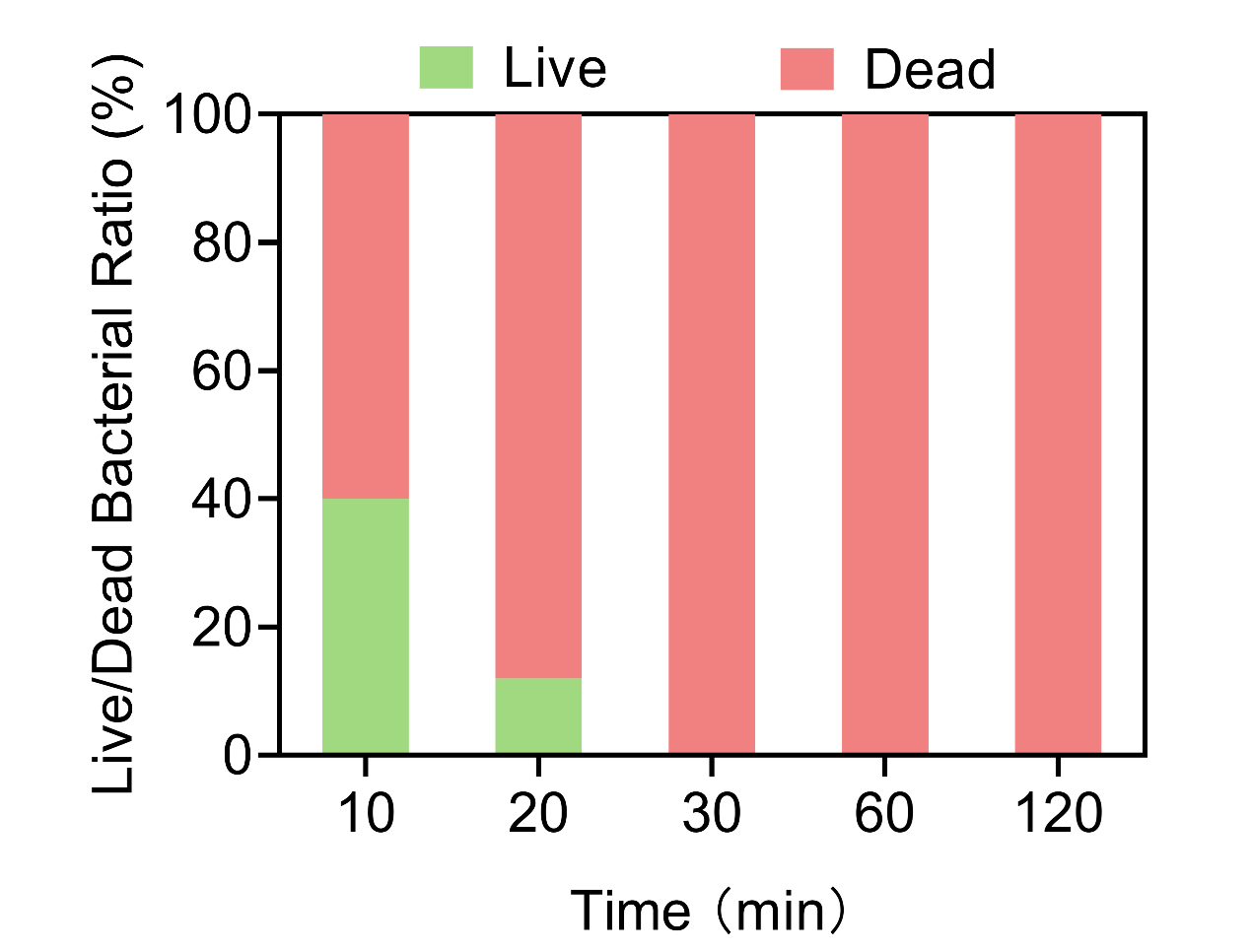
**Figure S8.** Statistical diagram of semi-quantitative fluorescence analysis of *E. coli* treated with 2 × MIC of C_6_PLL_10_-Gua for different.


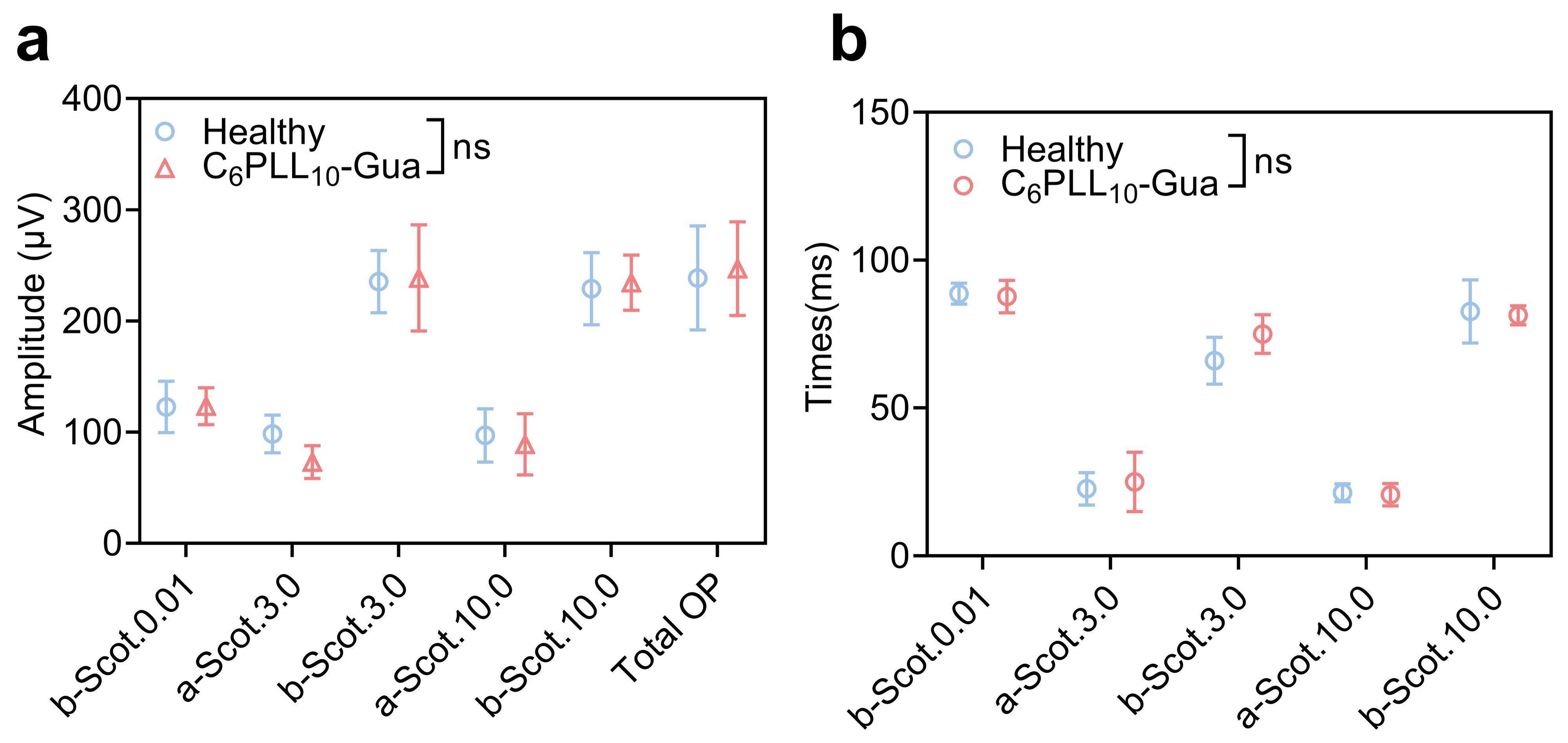


**Figure S9**. Electrophysiological (ERG) evaluations of retinal function in various groups. a) The amplitude in different ERG; b) The latency in different ERG.


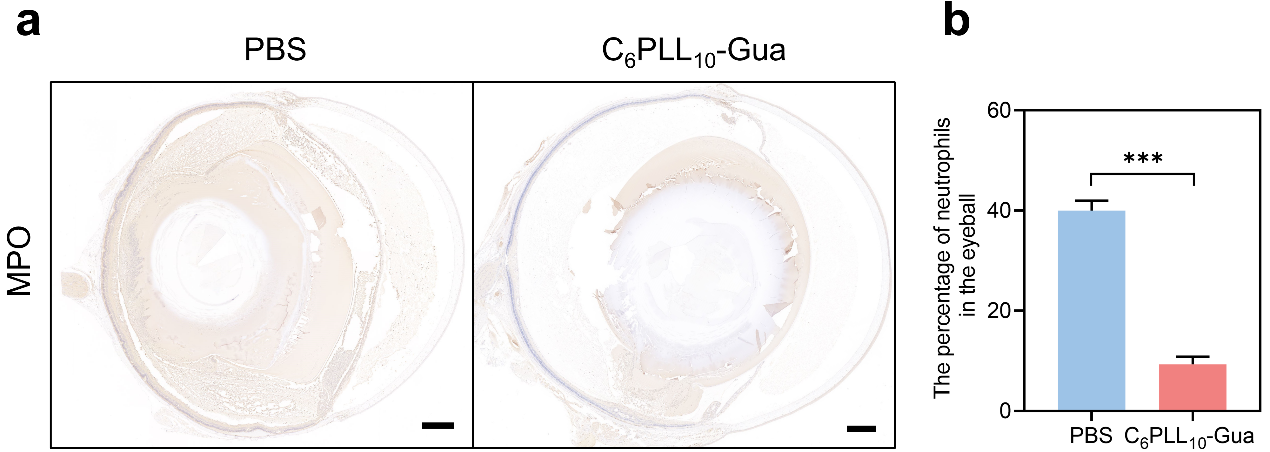


**Figure S10.** a) Immunohistochemical staining analysis of MPO in the ocular indicated the obviously decrease of the cytokine after the PBS or C_6_PLL_10_-Gua treatment. Scar bar: 500 µm. b) The percentage of neutrophils in the eyeball treated with diffferent treatment (****p* < 0.001).


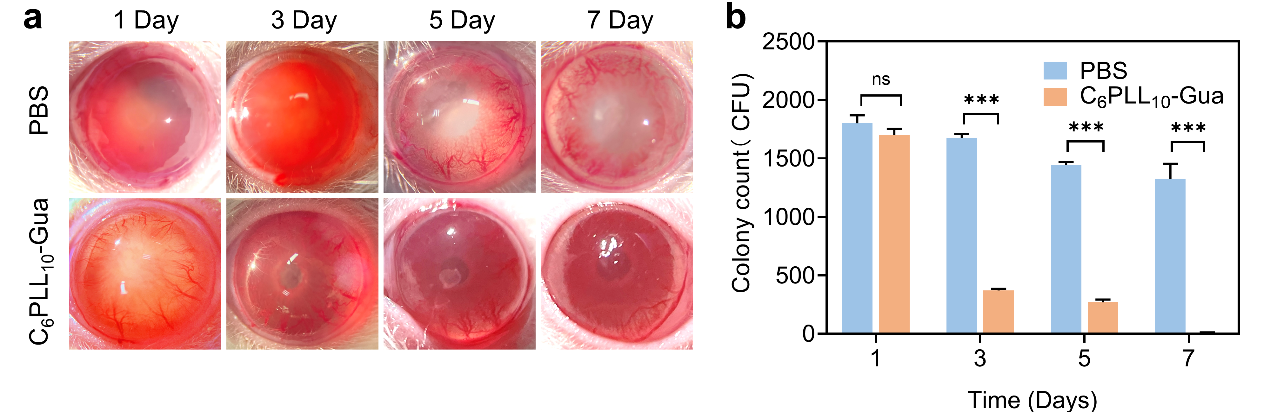


**Figure S11.** a) Typical Eye slit-lamp photos on days 1, 3, 5, and 7 in different treatments. b) Bacterial colony count of rat eyes. The colony count of endophthalmitis on days 1, 3, 5, 7 of C_6_PLL_10_-Gua group was lower than that in PBS group, and the difference was statistically significant on days 3, 5, 7 (****p*＜ 0.001). There was no significant difference on day 1 (^ns^*p*＞0.05).


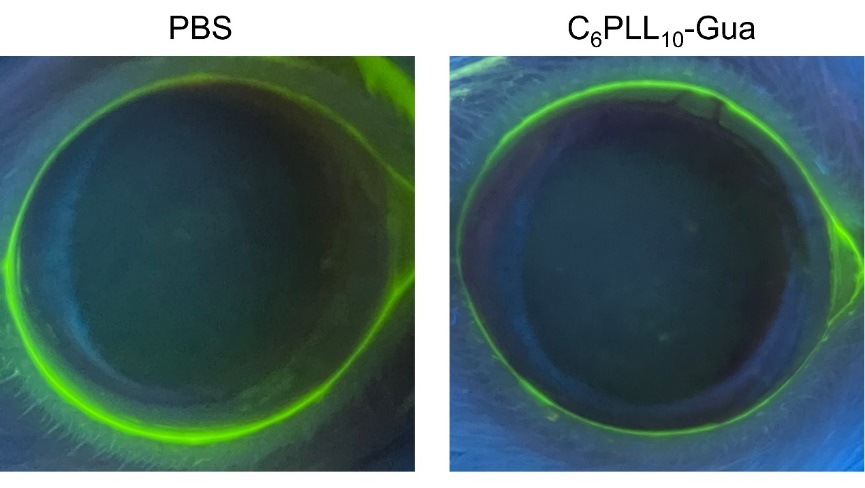


**Figure S12.** Representative images of fluorescein cornea staining of rat eyes after treatment with PBS or C_6_PLL_10_-Gua for seven consecutive days.


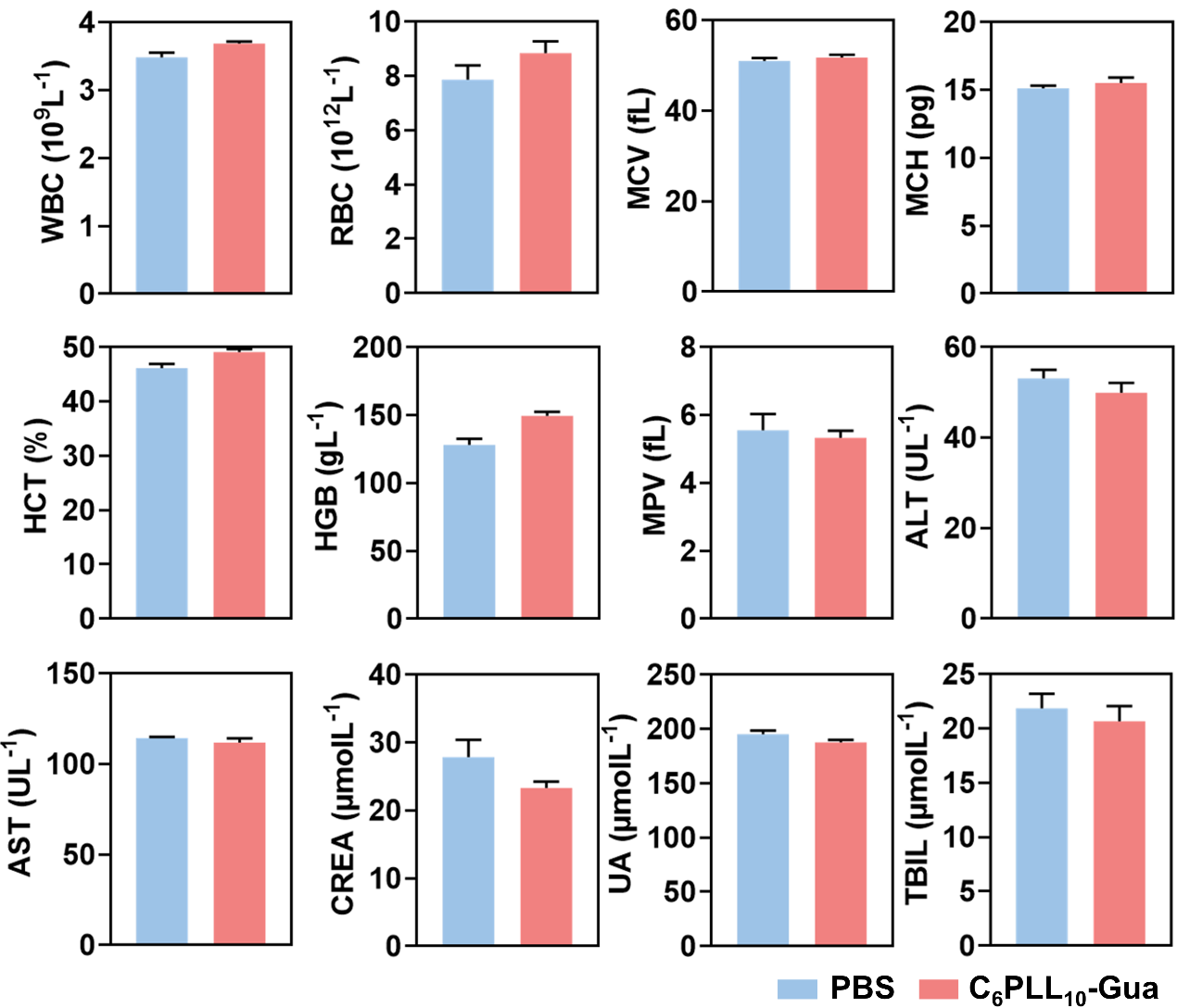


**Figure S13.** Major blood biochemical indexes of rat treated with PBS, methicillin or C_6_PLL_10_-Gua. Blood samples were taken at day 7 after initiating treatment. Error bars denote SD over three rats in each group.


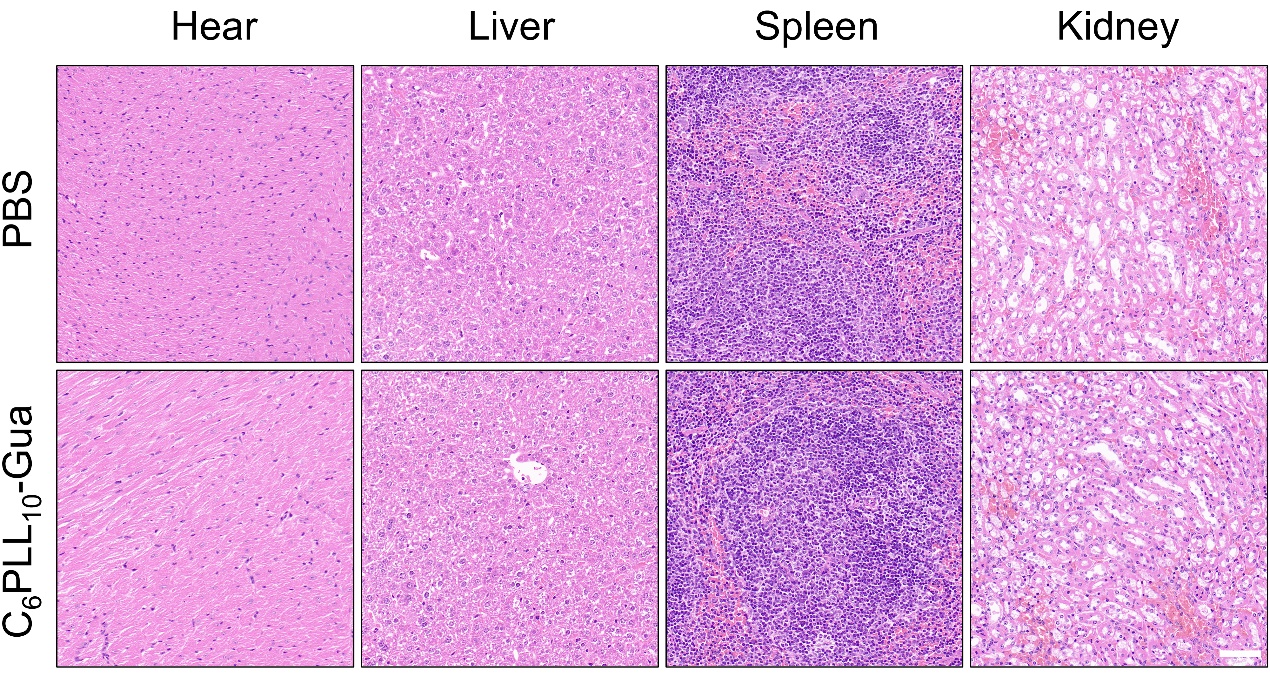


**Figure S14.** H&E staining analysis of main organs (heart, liver, spleen and kidney) of the rat in PBS and C_6_PLL_10_-Gua treated groups. (Scale bar: 50 μm).

**Table S1**. Characterization of C_x_-PLL_n_ and C_x_-PLL_n_-Gua

| Entry | Polymer | Feeding  [M]/[I]^a^ | DP^[b]^ | *M_n_^[c]^* [kg/mol] | *Đ^[c]^* |
| --- | --- | --- | --- | --- | --- |
| 1 | C_6_PLL_10_ | 10 | 13 | 1.3 | 1.03 |
| 2 | C_6_PLL_30_ | 30 | 28 | 3.5 | 1.24 |
| 3 | C_6_PLL_10_-Gua | 10 | 13 | 1.7 | 1.03 |
| 4 | C_6_PLL_30_-Gua | 30 | 29 | 5.7 | 1.18 |

^a^ [M] is the ε-benzyloxycarbonyl-L-lysine *N*-carboxyanhydride (ZLL-NCA), [I] represent the initiator.

^b^ Determined by ^1^H NMR in D_2_O.

^c^ GPC characterization of the polymers. 0.5M acetic acid and sodium acetate solution was used as solvent, calibrated with polyethylene glycol (PEG) standard.

**Table S2.** The MIC values of synthetic polymers against different bacteria

| Polymer | MICs (μg/mL)  *S. warner S. epider* *E. faecalis* | | |
| --- | --- | --- | --- |
| C_6_PLL_10_ | 12.5 | 12.5 | 12.5 |
| C_6_PLL_30_ | 32 | 32 | 25 |
| C_6_PLL_10_-Gua | 8 | 4 | 12.5 |
| C_6_PLL_30_-Gua | 64 | 32 | 25 |

**Reference**

[1] Du Y, Yan W, Lian H, Xiang C, Duan L, and Xiao C, 2,2'-Dithiodisuccinic acid-stabilized polyion complex micelles for pH and reduction dual-responsive drug delivery. J Colloid Interface Sci, 2018. 522: p. 74-81.

[2] Wan P, Wang Y, Guo W, Song Z, Zhang S, Wu H, Yan W, Deng M, and Xiao C, Low-Molecular-Weight Polylysines with Excellent Antibacterial Properties and Low Hemolysis. ACS Biomater Sci Eng, 2022. 8(2): p. 903-911.

[3] Shen W, Zhang Y, Wan P, An L, Zhang P, Xiao C, and Chen X, Antineoplastic Drug-Free Anticancer Strategy Enabled by Host-Defense-Peptides-Mimicking Synthetic Polypeptides. Adv Mater, 2020. 32(36): p. e2001108.

[4] Yin R, Wan P, Guo Z, Yi X, Zhang P, Shen W, Chen L, Xiao C, and Chen X, Enzyme-responsive oncolytic polypeptide for tumor therapy. Acta Biomater, 2024. 181: p. 415-424.

[5] Alfaar A S, Stürzbecher L, Diedrichs-Möhring M, Lam M, Roubeix C, Ritter J, Schumann K, Annamalai B, Pompös I M, Rohrer B, Sennlaub F, Reichhart N, Wildner G, and Strauß O, FoxP3 expression by retinal pigment epithelial cells: transcription factor with potential relevance for the pathology of age-related macular degeneration. J Neuroinflammation, 2022. 19(1): p. 260.

[6] Shang W, Sun Q, Zhang C, Liu H, Yang Y, Liu Y, Gao W, Shen W, and Yin D, Drug in Therapeutic Polymer: Sinomenine-Loaded Oxidation-Responsive Polymeric Nanoparticles for Rheumatoid Arthritis Treatment. ACS Appl Mater Interfaces, 2023. 15(40): p. 47552-47565.
